# Maternal immunity shapes biomarkers of germinal center development in HIV‐exposed uninfected infants

**DOI:** 10.3389/fimmu.2024.1443886

**Published:** 2024-09-12

**Authors:** Li Yin, Guglielmo M. Venturi, Richard Barfield, Bernard M. Fischer, Julie J. Kim-Chang, Cliburn Chan, Kristina De Paris, Maureen M. Goodenow, John W. Sleasman

**Affiliations:** ^1^ Molecular HIV Host Interactions Section, National Institute of Allergy and Infectious Diseases, Bethesda, MD, United States; ^2^ Division of Allergy and Immunology, Department of Pediatrics, Duke University School of Medicine, Durham, NC, United States; ^3^ Department of Biostatistics and Bioinformatics, Duke University School of Medicine, Durham, NC, United States; ^4^ Department of Microbiology and Immunology, School of Medicine, University of North Carolina at Chapel Hill, Chapel Hill, NC, United States; ^5^ Institute of Global Health and Infectious Diseases, School of Medicine, University of North Carolina at Chapel Hill, Chapel Hill, NC, United States

**Keywords:** HIV, pregnancy, immune development, A proliferation-inducing ligand (APRIL), macrophage, lymphoid germinal centers, HIV-exposed uninfected (HEU) infants, HIV-unexposed uninfected (HUU) infants

## Abstract

**Introduction:**

HIV-exposed uninfected (HEU) infants exhibit elevated pro-inflammatory biomarkers that persist after birth. However, comprehensive assessments of bioprofiles associated with immune regulation and development in pregnant women with HIV (PWH) and HEU infants has not been performed. Maternal immunity in PWH may be imprinted on their HEU newborns, altering immune bioprofiles during early immune development.

**Methods:**

Cryopreserved paired plasma samples from 46 HEU infants and their mothers enrolled in PACTG 316, a clinical trial to prevent perinatal HIV-1 transmission were analyzed. PWH received antiretrovirals (ARV) and had either fully suppressed or unsuppressed viral replication. Maternal blood samples obtained during labor and infant samples at birth and 6 months were measured for 21 biomarkers associated with germinal centers (GC), macrophage activation, T-cell activation, interferon gamma (IFN-γ)-inducible chemokines, and immune regulatory cytokines using Mesoscale assays. Pregnant women without HIV (PWOH) and their HIV unexposed uninfected (HUU) newborns and non-pregnant women without HIV (NPWOH) served as reference groups. Linear regression analysis fitted for comparison among groups and adjusted for covariant(s) along with principal component analysis performed to assess differences among groups.

**Results:**

Compared with NPWOH, PWOH displayed higher levels of GC, macrophage, and regulatory biomarkers. PWH compared to PWOH displayed elevated GC, T cell activation, and IFN-γ-inducible chemokines biomarkers at delivery. Similar to their mothers, HEU infants had elevated GC, macrophage, and IFN-γ-inducible chemokines, as well as elevated anti-inflammatory cytokines, IL-10 and IL-1RA. Across all mother/newborn dyads, multiple biomarkers positively correlated, providing further evidence that maternal inflammation imprints on newborn bioprofiles. By 6 months, many HEU biomarkers normalized to levels similar to HUU infants, but some GC and inflammatory biomarkers remained perturbed. Bioprofiles in PWH and HEU infants were similar regardless of the extent of maternal viral suppression by ARV.

**Conclusions:**

GC immune pathways are perturbed in HEU newborns, but immune regulatory responses down regulate inflammation during early infancy, indicating a transient inflammatory effect. However, several GC biomarkers that may alter immune development remain perturbed.

## 1 Introduction

HIV-exposed uninfected infants and children have increased morbidity and mortality associated with susceptibility to severe infections and neurodevelopmental delay (1–6). While the causes of these morbidities are complex, impaired immune development contributes to the adverse outcomes throughout childhood (7–13). There is increasing evidence that HIV-associated immune dysregulation in pregnant women with HIV (PWH) adversely impacts their HIV-exposed uninfected (HEU) infants (1, 5, 14–16).

Pregnancy, in the absence of HIV infection, is associated with profound changes in maternal immunity that is tightly regulated from early fetal implantation to the time of delivery (17, 18). To assure maternal tolerance toward fetal tissues and to protect the fetus from infection, key shifts in immunity and immune bioprofiles occur throughout the course of gestation (19–21). By the time of labor and delivery, the fetal/maternal interface is skewed toward a pro-inflammatory state primarily mediated by activated monocytes/macrophages within the placenta (20, 22). Macrophage activation in late pregnancy is countered by enhanced immune regulatory pathways including T regulatory cells (23, 24). Immune dysregulation of the fetal/maternal interface is associated with adverse pregnancy outcomes, such as pre-term birth and preeclampsia (20, 24, 25). In PWH, further HIV-associated perturbations of maternal pro-inflammatory pathways are thought to contribute to an increased incidence of adverse pregnancy outcomes in HEU newborns (26, 27).

In HIV-unexposed and uninfected (HUU) newborns, germinal center (GC) architecture consist of macrophages, dendritic cells, T cells, and B cells that are under-developed compared to older children (21). Within GC of HUU newborns there is a paucity of T follicular helper cells associated with decreased expression of Th1 cytokines and functional immaturity of follicular dendritic cells (28–30). Critical for immune priming in early life, GC components shape effective vaccine responses in infants by directing immunoglobulin class switch via IL-21, CD40/CD40L, production of B cell developmental cytokines such as APRIL and BAFF, and cognate interactions between T cells and B cells (31–35). Plasma GC biomarkers detected in cord blood can predict vaccine responses in later infancy in HUU infants (36).

Compared to HUU newborns, HEU newborns and infants exhibit elevated pro-inflammatory bioprofiles similar to maternal profiles, including few studies directly comparing PWH and pregnant women without HIV (PWOH) mother/baby dyads (9, 15, 37). Over the first 6 months of life and beyond, HEU infants display persistent monocyte and antigen presenting cell activation, impaired T cell responses, and upregulated T regulatory cell function (38, 39). Some of these previous studies have examined GC biomarkers such as IL-21 but there has not been a comprehensive assessment of bioprofiles associated with GC development that included key factors such as APRIL, BAFF, and CD40L (15).

The current study is based on the hypothesis that the maternal immune milieu in PWH can imprint early immune bioprofiles in HEU newborns and infants. To test this hypothesis and to extend the scope of previous investigations of immunity in HEU newborns and infants, a panel of plasma biomarkers reflecting T cell activation and differentiation, such as sCD27, IL-17, IL-22, and IL-2, and interferon (IFN)-induced chemokines, combined with biomarkers of GC development was developed to assess a broad array of biomarkers associated with early immune development. Plasma samples obtained from PWH and their HEU infants enrolled in Pediatric AIDS Clinical Trial Group (PACTG) 316, the largest United States clinical trial to prevent perinatal HIV transmission, were evaluated (40). Maternal samples were assessed during labor along with term HEU infants at birth and 6 months. A cohort of PWOH and their term HUU infants were included for comparison as well as a longitudinal cohort of HUU infants from birth to 6 months. This novel study fills important gaps in the understanding of the effect of pregnancy and HIV infection on immune networks and how maternal immunity shapes immune development in infants.

## 2 Materials and methods

### 2.1 Study cohort and plasma sample collection

Plasma samples were selected from biorepositories established to enhance retrospective analysis of archived blood samples from study participants enrolled in clinical trials. Archived samples from PWH and their HEU infants were obtained from Biomedical Research Institute Repository (Rockville, MD) that were stored from participants enrolled in PACTG 316, a nevirapine study for the prevention of HIV transmission from mothers to babies (ClinicalTrials.gov ID: NCT00000869). This was an international, multicenter, randomized, double-blinded, placebo-controlled phase III clinical trial that enrolled a total of 1,506 mother/infant dyads between 1997 and 2000 (40). All PWH received antiretrovirals (ARV) according to the standard of medical care at the time of enrollment and were randomized to receive nevirapine or placebo as a single dose for the mother during labor, and for the newborn immediately following delivery. For the current study, 46 PWH and their HEU infants from clinical sites within the United States, primarily from sites in the northeastern and southeastern regions were included. Based on the protocol, maternal plasma samples were drawn from PWH during labor (within 2 days prior to delivery); two exceptions were samples drawn before labor at 11 and 13 days prior to delivery. PWH were excluded from selection if they had active infection, history of AIDS defining illnesses, or CD4 T cell counts < 200 cells/µl. Pre-term newborns (gestation < 37 weeks), were excluded because prematurity alters immune bioprofiles (41–44). Half of the PWH (n = 23) achieved viral suppression (VS) on ARV, defined as plasma viral load (VL) ≤ 400 HIV-1 RNA copies/mL at the time of sampling, while the other half (n = 23) achieved partial viral suppression (virally non-suppressed, VNS) with plasma VL > 400 HIV-1 RNA copies/mL (range 417 to 63,444 copies/mL). All HEU newborn blood samples were obtained as cord blood (CB) with a second venous blood sample obtained at 6 months of age. HEU infants were all term infants (gestation > 37 weeks), per protocol, delivered vaginally or by caesarian delivery, and all were exclusively formula fed to prevent transmission of HIV by breast milk (**Table 1**).

**Table 1:** Demographics and clinical characteristics of study participants.

		Study groups						
		NPWOH (n=21)	PWOH (n=18)	HUU (n=50)^a^	PWH-VS (n=23)	HEU-MVS (n=23)	PWH-VNS (n=23)	HEU-MVNS (n=22)^b^
**Time point**		Entry	Labor^c^	Birth & 6m	Labor^c^	Birth & 6m	Labor^c^	Birth & 6m
**Women’s age (year)^d^**		23 (20-30)	28 (23-31)	NA	26 (23-33)	NA	26 (23-30)	NA
**Self-reported as African Americans**		48%	17%	16%	44%		52%	
CD4 T cell count (cells/μL)^d,^*		ND	ND	ND	640 (433-798)	ND	440 (259-530)	ND
**Viral load (HIV-1 RNA copies/mL plasma)^d^**		NA	NA	NA	< 400^e^	NA	2,881 (1,505–22,948)	NA
**ARV during** pregnancy**	**ZDV alone**	NA	NA	NA	1 (4%)	NA	10 (44%)	NA
	**NRTI combo**	NA	NA	NA	9 (39%)	NA	7 (30%)	NA
	**NRTI combo + PI**	NA	NA	NA	13 (57%)	NA	6 (26%)	NA
**ARV to mother** **during labor**	**Nevirapine** **single dose**	NA	NA	NA	11 (48%)		13 (57%)	
**Pregnancy**		NA	NA	Term^f^	NA	Term^f^	NA	Term^f^
**Baby sex**	**Male**	NA	NA	50%	NA	39%	NA	55%
	**Female**	NA	NA	50%	NA	61%	NA	46%
Baby birth weight (kg)^d,^*		NA	NA	3.6 (3.1–3.9)	NA	3.0 (2.8–3.4)	NA	3.0 (2.8-3.4)
**Mode of delivery**	**Vaginal**	NA	100%		65%		46%	
	**Cesarean section**	NA	0%		35%		55%	
**Infant feeding** **method**	**Breast fed**	NA	NA	59%	NA	0%	NA	0%
	**Formula fed**	NA	NA	16%	NA	100%	NA	100%
	**Mixed fed**	NA	NA	25%	NA	0%	NA	0%

NPWOH, non-pregnant women without HIV; PWOH, pregnant women with HIV; PWH-VS, pregnant women with HIV virally suppressed; HEU-MVS, HIV-exposed uninfected born to virally suppressed mother; PWH-VNS, pregnant women with HIV virally non-suppressed; HEU-MVNS, HIV-exposed uninfected born to virally non-suppressed mother; ARV, antivirals; ZDV, zidovudine; NRTI, nucleotide reverse transcriptase inhibitor; PI, protease inhibitor; ND, not determined; NA, not applicable.

^a^all 50 HUU babies have birth cord blood samples, including 32 HUU who also have 6 month venous blood samples; 18 HUU have maternal data; ^b^twin newborns were excluded from the analysis; ^c^blood sample obtained during labor within 2 days before delivery; ^d^Median (25% - 75% quartile range); ^e^undetectable HIV-1 viral load < 400 HIV-1 RNA copies/mL plasma; ^f^term pregnancy is defined as delivery between 37 weeks 0 days and 41 weeks 6 days (44)

*p < 0.05 by Mann-Whitney U test (comparing CD4 T-cell between two PWH groups or birth weight between HUU and HEU babies); **p < 0.05 by Fisher’s exact test (comparing ARV during pregnancy between two PWH groups).

Samples from healthy women and infants in the current study were obtained from 50 HUU newborns and infants, 18 PWOH, and 21 non-pregnant women without HIV (NPWOH), available in the Duke Pediatric Immunology Biorepository, established for future studies involving healthy mothers and infants, and approved by the Institutional Review Board of Duke University (IRB#: Pro00056028). Stored newborn and infants’ samples were obtained under a protocol, the effect of breast feeding on immunologic priming in young infants (ClinicalTrials.gov Identifier NCT02568579) and collected from sites in Florida and North Carolina between 2011 and 2017. All samples came from term deliveries (gestation > 37 weeks), delivered vaginally with paired PWOH and newborn CB samples from 18 mother/newborn dyads. Among the 50 newborns, 32 were followed over the first 6 months of life and were either breast fed, formula fed, or a mixture of breast and formula feeding (**Table 1**). Maternal plasma samples from PWOH collected during labor (within 2 days before delivery). Additional single time point plasma samples from 21 NPWOH were selected for comparison to PWOH to identify the influence of pregnancy on individual biomarkers. These *participants were self-declared healthy individuals who were enrolled as the control group for Substance Use and Immunity in HIV+ Adolescents by Systems Biology* (ClinicalTrials.gov Identifier NCT00683579) between 2011 and 2021. Details for this cohort have been previously described (45).

The current study design was formulated based on a 2019 request from the Eunice Kennedy Shriver National Institute of Child Health and Human Development (NICHD) for exploratory/developmental R21 research proposals (RFA-HD-19-018). The use of archived samples was approved by NICHD counsel and a research proposal, *Impact of maternal HIV infection on immune priming of their uninfected infants*, was peer reviewed by an NIH scientific review panel with the notice of award issued on April 14, 2020. Under a separate application (NWCS 629), a request for samples was submitted and approved by the International Maternal Pediatric Adolescent AIDS Clinical Trials Network (IMPAACT) on August 6, 2020, and the specimens were released for study. All PWH and HEU samples and clinical information obtained from PACTG 316 participants, PWOH, HUU, and NPWOH were de-identified. All participants or legal guardians provided signed informed consent. Ethical review and approval for use of the samples involving human participants was provided by Duke University Institutional Review Board: Pro00050219 - *Effects of Breast Feeding on Immunologic Priming in Young Infants and Pro00106528 - Impact of Maternal HIV Infection on Immune Priming of Their Uninfected Infants*, Office of Human Subject Research (OHSR) of National Institute of Health (exemptions 13412, 13413 and 13414).

### 2.2 Biomarkers

Double spun frozen plasma aliquots from whole blood were used in batch analysis. Twenty-three biomarkers associated with B cell and GC development (APRIL, BAFF, sCD40L, IL-21), macrophage activation (sCD14, sCD163), T cell activation and differentiation (sCD27, IFN-γ, IL-17A, IL-22, IL-2, IL-4, and IL-23), IFN-γ-inducible chemokines (CXCL9/MIG, CXCL10/IP-10), immune activation chemokines (CCL4/MIP-1β, CCL5/RANTES, CXCL8/IL-8), inflammatory cytokines (TNF-α, IL-1β, IL-6), and immune regulatory cytokines (IL-10, IL-1RA) were assessed. APRIL was measured with a magnetic bead based Luminex singleplex assay (Bio-Rad, Hercules, CA), other biomarkers were quantified by singleplex (sCD14 and CCL5/RANTES), or multiplex (all other analytes) assays, using the Meso Scale Diagnostics (Rockville, MD, USA) platform, according to the manufacturer’s instructions. IL-4 and IL-23 were excluded from further analyses because more than 40% of the samples had values below the limit of detection for the assays. All values for the remaining 21 plasma biomarker concentrations are shown in **Supplementary Tables 1**, **2**.

### 2.3 Study design

**Figure 1:**
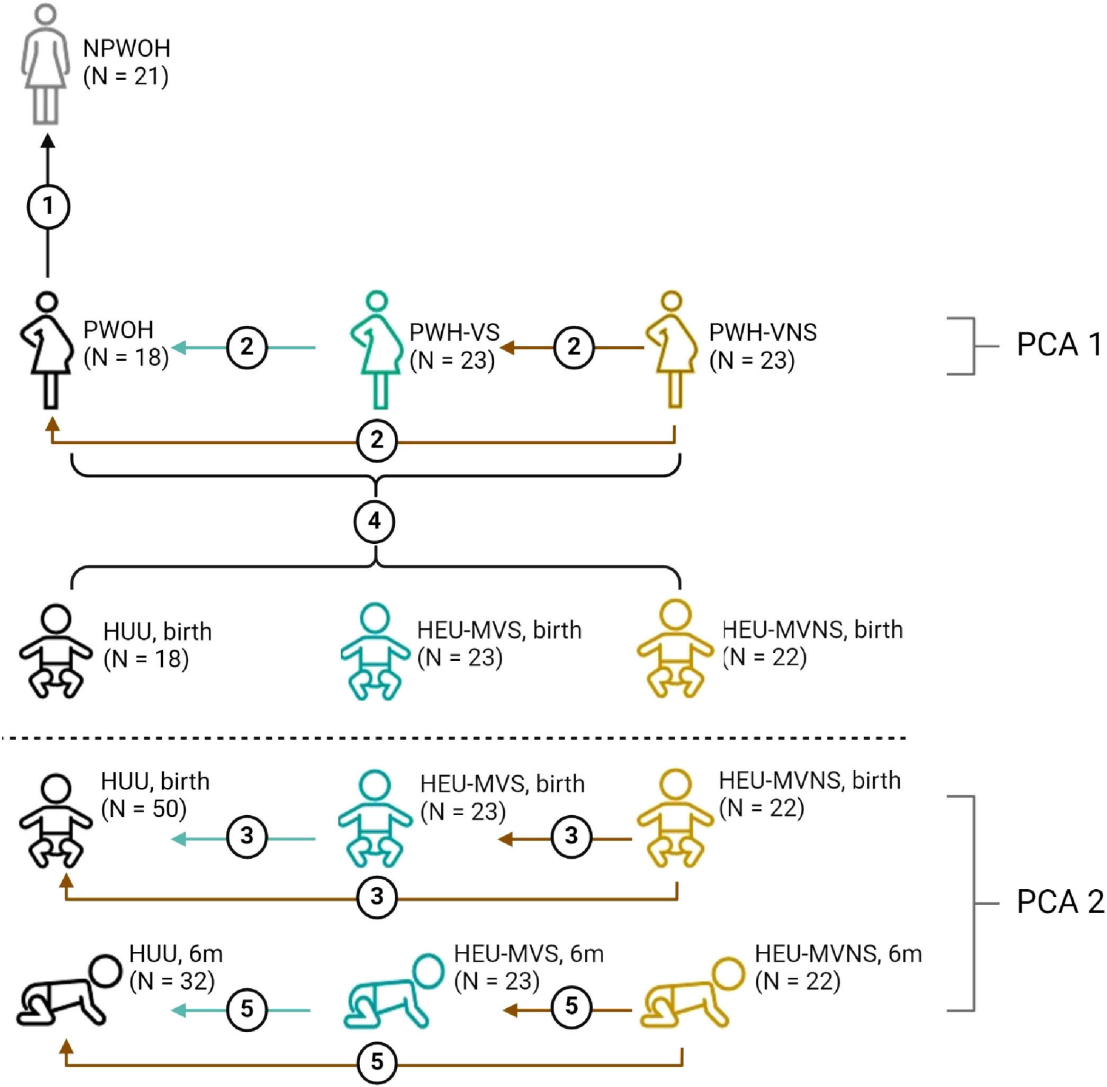
Overview of biomarker analyses plans across study groups. Analyses 1-3 and 5 compare biomarker concentrations; analysis 4 correlates biomarker concentrations between mother and baby dyads; PCA1 demonstrates how the pregnant women clustered relative to all biomarkers; PCA2 illustrates how the infants clustered relative to all biomarkers at birth and at 6 months of life; dotted line divides studies involving women, and studies involving infants only. Analysis 1, compare NPWOH with PWOH; Analysis 2, compare among PWOH, PWH-VS, and PWH-VNS; Analysis 3, compare among HUU, HEU-MVS, and HEU-MVNS newborns at birth; Analysis 4, correlation between all pregnant women (PWOH, PWH-VS, and PWH-VNS) with all their newborns (HUU, HEU-MVS, and HEU-MVNS); Analysis 5, compare among HUU, HEU-MVS, and HEU-MNS infants at 6 months of age. The number of participants in each group is shown in parentheses. The graphic was created with BioRender.com. NPWOH, non-pregnant without HIV; PWOH, pregnant without HIV; PWH-VS, pregnant with HIV virally suppressed; PWH-VNS, pregnant with HIV virally non-suppressed; HUU, HIV-unexposed uninfected; HEU-MVS, HIV-exposed uninfected born to PWH-VS; HEU-MVNS, HIV-exposed uninfected born to PWH-VNS. PCA, principal component analysis.

Plasma biomarker concentrations were compared across four distinct groups: 1) NPWOH and PWOH, 2) PWOH and PWH, 3) HUU and HEU newborns, and 4) HUU and HEU infants at 6 months. The study design is outlined in **Figure 1**. The first analysis compared PWOH (n = 18) and NPWOH (n = 21) to determine how third trimester pregnancy influences immune bioprofiles in the absence of HIV infection. Second, comparison of PWOH (n = 18) and PWH (n = 46), including PWH-VS (n = 23) and PWH-VNS (n = 23) measured the impact of suppressed HIV infection on maternal bioprofiles near the time of delivery. Third, to determine how bioprofiles of HIV exposed-uninfected newborns differ from unexposed newborns, HUU and HEU newborns whose mothers’ viral replication were suppressed (HEU-MVS) or not suppressed (HEU-MVNS) were analyzed. Fourth, direct comparisons at birth between mother/baby dyads (each mother including PWOH, PWH-VS, and PWH-VNS vs her newborn including HUU, HEU-MVS, and HEU-MVNS) to determine if biomarkers in newborns reflect the biomarkers in their mothers. Fifth, comparison among 6-month-old HUU and HEU infants (HEU-MVS and HEU-MVNS) to determine how bioprofiles of HEU newborns differ from HUU at 6 months of life and assess the changes in bioprofiles over time.

### 2.4 Data analysis

Biomarker concentrations were log_10_ transformed with values below the lower detection limit placed at one-half the assay lower limit for analysis. Statistical analyses were performed within R. PCA was performed using the prcomp function within R. Linear regression was performed for all analyses except when observations were at a lower limit of detection (left censored data), in which case Tobit regression was performed. Mother’s age was adjusted for all analysis (**Figure 1**), while babies’ sex was adjusted relative to their biomarkers (**Figure 1**, analyses 3-5). In the analysis of biomarker correlation between pregnant women, or mother/newborn dyads, adjustments were made for the pregnant women’s HIV and viral status (**Figure 1**, analyses 2 and 4). Multiple testing corrections were via Benjamini-Hochberg FDR (https://www.jstor.org/stable/2346101) with an unadjusted p-value less than 0.05 and an FDR corrected p-value less than 0.1 set as significant. Pairwise comparisons among three groups of mothers (PWOH, PWH-VS, PWH-VNS) or babies (HUU, HEU-MVS, HEU-MVNS newborns or infants) were performed with significance set at p < 0.0167 (p < 0.05/3) using Bonferroni correction.

Selection of archived plasma samples from multiple cohorts collected at different times and locations results in inherent confounders within the data sets. To address this, we performed a sensitivity analysis on infant biomarkers that were associated with mother’s HIV status focusing on the mode of delivery, randomization arm for PACTG 316, and feeding methods for the 6-month analysis. We reran the model testing for significant biomarkers at birth and six months without adjustment for any confounders on varying subgroups of infants. To assess the role of mode of delivery and PACTG randomization arm, we compared the HUU to a subgroup of HEU who were randomized to placebo and had vaginal delivery. We collapsed the two HEU newborns from the PWH-VS and PWH-VNS groups together due to the small sample size, and no differences between these two HEU groups were observed. To assess the impact of feeding method on the 6-month analysis, we compared the HEU infants at 6 months to HUU infants who received exclusively formula, or a mix of formula and breast fed. Results of the sensitivity effect estimates were compared to the results obtained for the entire HEU and HUU cohorts. PCA was also performed in the HEU group via the prcomp function within R to assess for any clear patterns by these confounders. For the PCA, values at the lower detection limit placed at one-half the lower limit and all data was log_10_ transformed. Missing data was mean imputed solely for the PCA.

## 3 Results

Overall, 17% of PWOH self-identified as African American, while about half of NPWOH and PWH were African American. Women’s age was similar across the groups (**Table 1**). Among 46 PWH at delivery, CD4 T cells counts were higher in PWH-VS [median 640 (range 433 – 798 cells/µL)], compared to PWH-VNS [median 440 (range 259 – 530 cells/µL)] (p = 0.0039). While all PWH received ARV during pregnancy, half of PWH had undetectable plasma VL (≤ 400 HIV-1 RNA copies/mL), and half had detectable plasma viral levels [median 2,881 (range 1,505 – 22,948) HIV-1 RNA copies/mL]. ARV among PWH varied as a higher proportion of PWH-VS received nucleotide reverse transcriptase inhibitor (NRTI) in combination with a protease inhibitor (PI), while more PWH-VNS received zidovudine (ZDV) monotherapy (p = 0.006). Overall, 52% of PWH (24/46) were randomized to single dose nevirapine with similar distribution in VS [11/23 (48%)] and VNS [13/23 (57%)] PWH. The proportion of male and female babies was similar across all groups. Although the HEU newborns had lower birth weights compared to HUU (p <0.0001), all newborns included in the analysis were term deliveries, defined as > 37 weeks gestation (44). All 50 HUU newborns and the majority of HEU infants (26 of 46) were vaginally delivered (HEU-MVS, 65%; HEU-MVNS, 46%). Over the first 6 months of life all HEU infants were exclusively formula fed, while among HUU infants 16% were formula fed, 59% were breast fed, and 25% received mixed (formula and breast) feeding.

### 3.1 Influence of pregnancy on immune bioprofiles in women without HIV

**Figure 2:**
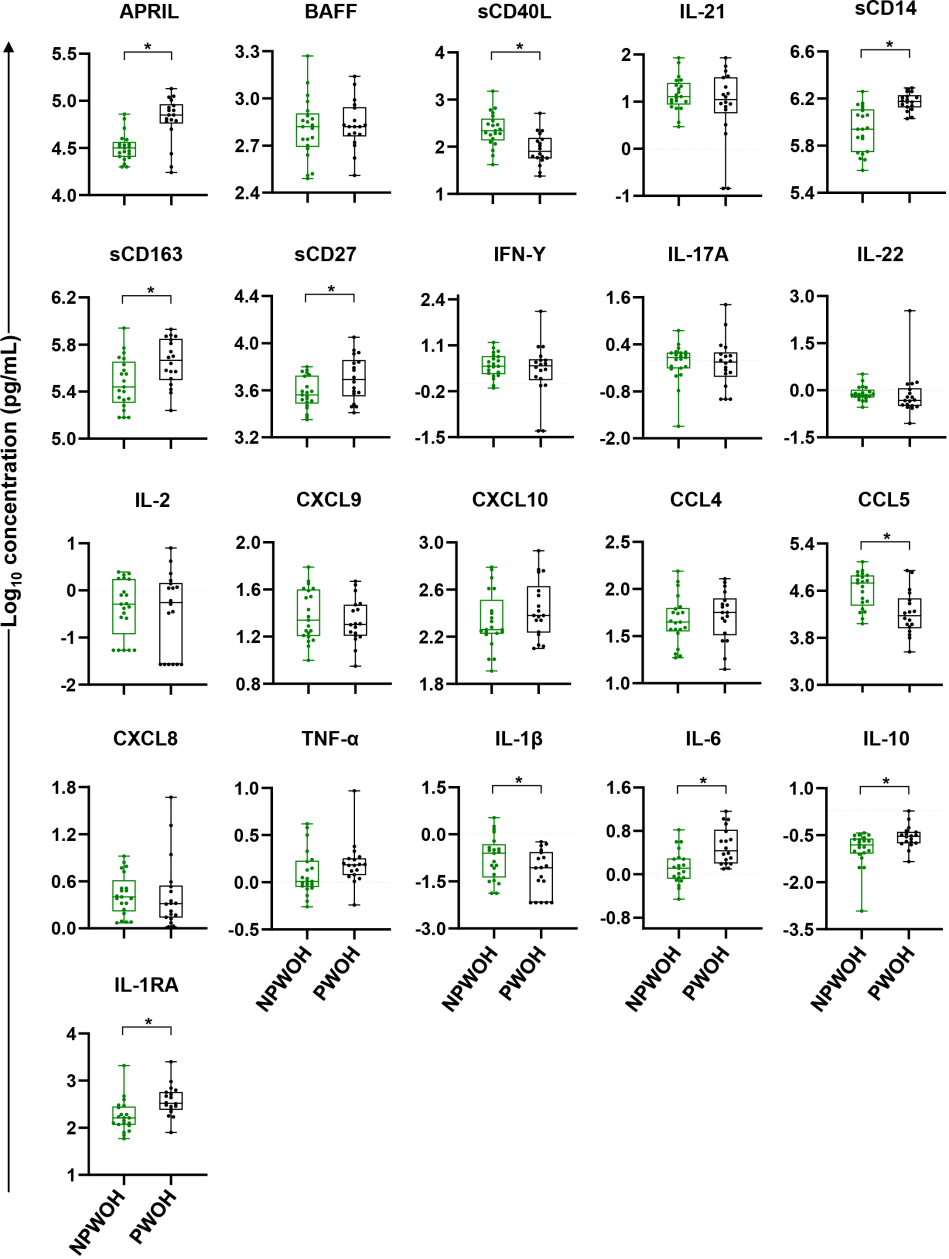
Comparison of plasma biomarker concentrations between non-pregnant and pregnant women without HIV. Comparisons between PWOH and NPWOH by plasma biomarkers sorted by association with germinal center development (APRIL, BAFF, sCD40L, IL-21), macrophage activation (sCD14, sCD163), T cell activation and differentiation (sCD27, IFN-γ, IL-17A, IL-22, IL-2), IFN-γ-inducible chemokines (CXCL9, CXCL10), immune activation chemokines (CCL4, CCL5, CXCL8), inflammatory (TNF-α, IL-1β, IL-6) and regulatory cytokines (IL-10, IL-1RA). The Y-axis shows log_10_ plasma biomarker concentrations (pg/mL). Adjusting for women’s age, linear regression was fitted to compare biomarker concentrations between PWOH (black dots) and NPWOH (green dots), and Tobit regression was used when data contained values below detection limit, including IL-21, IFN-γ, IL-17A, IL-2, IL-1β, and IL-10. Multiple testing correction was done via the Benjamini Hochberg FDR with significance assessed as an un-adjusted p < 0.05 and an FDR-adjusted p < 0.1 (shown as *). NPWOH, non-pregnant without HIV; PWOH, pregnant without HIV.

To identify immune bioprofiles associated with pregnancy, PWOH and NPWOH plasma biomarker concentrations were initially compared. PWOH had significantly higher concentrations of biomarkers associated with GC development (APRIL), macrophage activation and inflammation (sCD14, sCD163, and IL-6), T cell activation (sCD27), and immune regulation (IL-10 and IL-1RA), but lower concentrations of sCD40L, CCL5, and IL-1β (**Figure 2**). In contrast, no significant association was observed between pregnancy and plasma concentrations of GC cytokines BAFF and IL-21, T cell differentiation biomarkers (IFN-γ, IL-17A, IL-22, and IL-2), IFN-γ-inducible chemokines (CXCL9, and CXCL10), immune activation chemokines (CCL4 and CXCL8), or TNF-α. Taken together, third trimester pregnancy in the absence of HIV infection is associated with elevated pro-inflammatory bioprofiles, particularly sCD14 and sCD163, suggesting GC and macrophage activation.

### 3.2 Influence of HIV infection on immune bioprofiles during pregnancy

To assess the influence of HIV infection on pregnancy bioprofiles, biomarkers among PWH (PWH-VS and PWH-VNS) were compared with PWOH. Eleven biomarkers were similar among the groups of pregnant women independent of HIV status [including B cell developmental marker BAFF; macrophage activation biomarker sCD14, T cell markers IFN-γ, IL-17A, IL-22 and IL-2; immune activation markers CCL4 and CXCL8; inflammatory cytokine IL-6; and immune regulatory biomarkers IL-10 and IL-1RA] (**Supplementary Table 3**). In contrast, ten biomarkers showed significant associations across the three groups of pregnant women (**Supplementary Table 3**).

**Figure 3:**
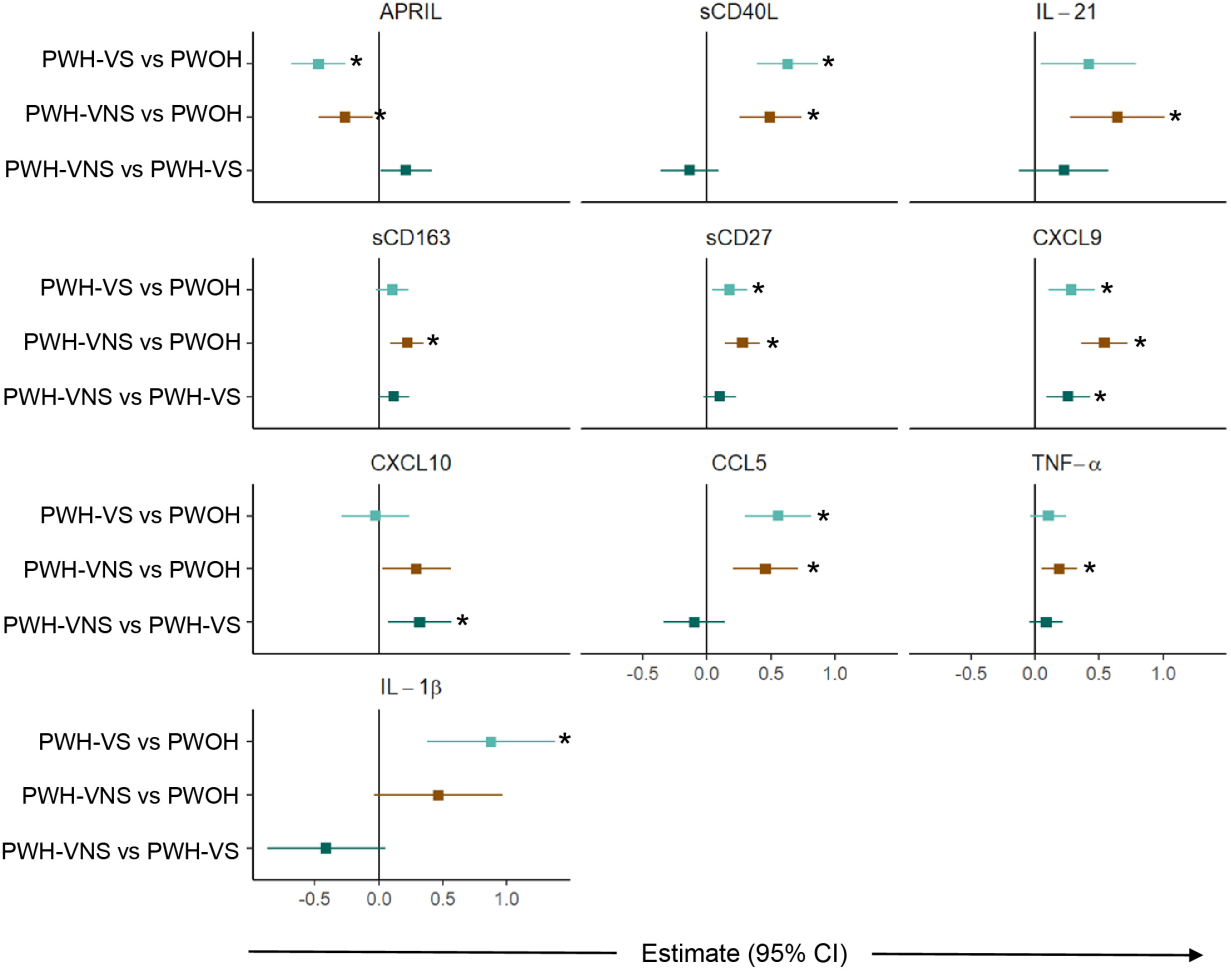
Comparison of plasma biomarker concentrations between pregnant women with and without HIV. Forest plot for 10 plasma biomarkers which’s concentrations differed significantly among PWOH, PWH-VS, and PWH-VNS, while the concentrations of the other 11 biomarkers were similar. Pairwise effect estimates and confident intervals are shown from a linear regression model, adjusting for woman’s age. Tobit regression was used when data contained values below detection limit, including APRIL, IL-21, CXCL10 and IL-1β. For significant biomarkers (un-adjusted p < 0.05 and FDR-adjusted p < 0.1), pairwise comparisons were performed with significance set at p < 0.0167 (p < 0.05/3 Bonferroni correction) and shown with (*). 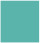 PWH-VS vs PWOH; 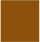 PWH-VNS vs PWOH; 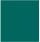 PWH-VNS vs PWH-VS. PWOH, pregnant without HIV; PWH-VS, pregnant with HIV virally suppressed; PWH-VNS, pregnant with HIV virally non-suppressed.

To further define significant bioprofiles among pregnant women, pairwise differences between the three groups were assessed (**Figure 3**). Relative to PWOH, PWH had lower concentrations of APRIL and higher concentrations of GC markers sCD40L and IL-21, macrophage activation marker sCD163, T cell activation and differentiation biomarker sCD27, IFN-γ-inducible cytokine CXCL9, and immune activation chemokines CCL5, TNF-α, and IL-1β. In general, independent of viral suppression status, PWH displayed similar concentrations of biomarkers with two exceptions: concentration of CXCL9 and CXCL10 were elevated among PWH-VNS compared to PWH-VS. These results indicate that at the time of labor, PWH had immune activation involving IFN-γ-inducible chemokines and T cell derived biomarkers with little difference between PWH-VNS and PWH-VS.

### 3.3 Immune activation bioprofiles in HEU newborns

Plasma biomarker concentrations in HEU and HUU newborns were tested for differences by their HIV exposure status. After adjusting for covariates of maternal age and infant sex, the regression analysis identified 15 biomarkers differing significantly across all groups. In contrast, six other biomarkers (BAFF, IFN-ү, IL-17A, IL-2, CXCL10 and CCL5) were similar among HEU-MVNS, HEU-MVS, and HUU (**Supplementary Table 4**).

**Figure 4:**
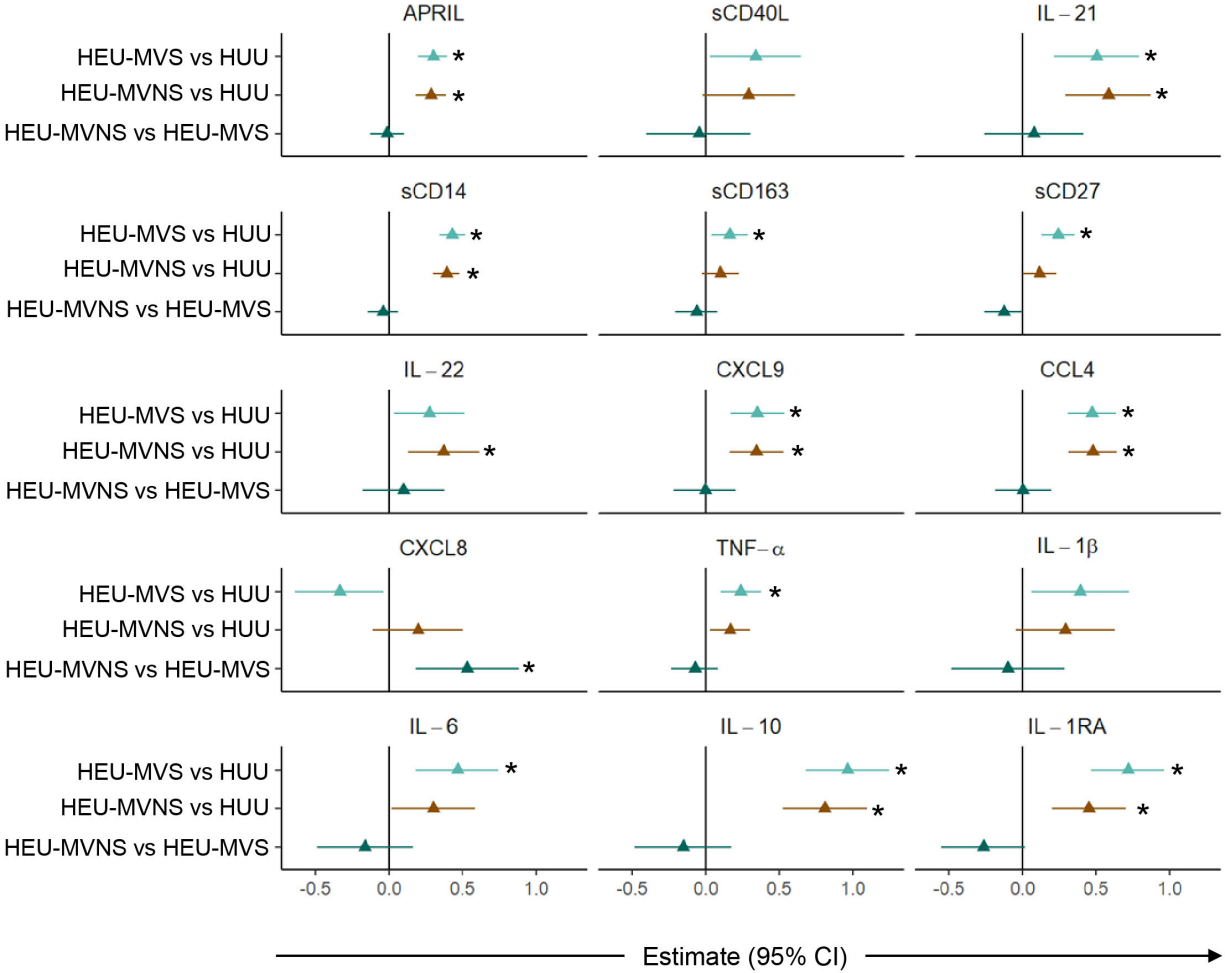
Comparison of plasma biomarker concentrations among newborns. Forest plots for 15 plasma biomarkers which’s concentrations differed significantly among HUU, HEU-MVS, and HEU-MVNS newborns, while the concentrations of the other 6 biomarkers were similar. Biomarker comparisons show pairwise effect estimates and confident intervals from a linear regression model, adjusting for mother’s age and newborn’s sex. Tobit regression was used when data contained values below detection limit, including IL-21, IL-22, IL-1β, and IL-10. For significant biomarkers (un-adjusted p < 0.05 and FDR-adjusted p < 0.1), pairwise comparisons were performed with significance set at p < 0.0167 (p < 0.05/3 Bonferroni correction) and shown with * 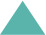 HEU-MVS vs HUU; 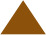 HEU-MVNS vs HUU; 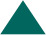 HEU-MVNS vs HEU-MVS. HUU, HIV-unexposed uninfected; HEU-MVS, HIV-exposed uninfected born to virally suppressed mother; HEU-MVNS, HIV-exposed uninfected born to virally non-suppressed mother.

Subsequent pairwise comparisons of HEU and HUU groups are shown in **Figure 4**. HEU newborns had higher biomarker concentrations distributed across the functional categories and included: GC biomarkers APRIL and IL-21, macrophage activation sCD14 and sCD163, T cell activation and differentiation biomarkers sCD27 and IL-22, IFN-γ-inducible chemokine CXCL9, immune activation chemokine CCL4, inflammatory cytokines TNF-α and IL-6, and immune regulatory cytokines IL-10 and IL-1RA. All biomarker concentrations were similar between HEU-MVS and HEU-MVNS with the exception of CXCL8 which was higher in HEU-MVNS than in HEU-MVS. sCD40L and IL-1β differed significantly among all three groups by regression analysis but were insignificant by pair-wise comparisons.

Sensitivity analyses performed on biomarkers that were significantly different between HEU and HUU to examine possible effects of confounding variables, particularly mode of delivery and randomization to the arm of PACTG 316. HUU newborns (n = 50) were compared to a subset of HEU newborns delivered vaginally and received placebo (n = 12). Comparison of this HEU subset to HUU newborns showed no change in the direction of effect estimates of the biomarkers, suggesting that nevirapine administration and caesarian delivery were not driving the biomarker results. Other adjustments were not performed due to the small sample size (**Supplementary Figure 1**).

### 3.4 Bioprofiles associations between mother/newborn dyads

**Figure 5:**
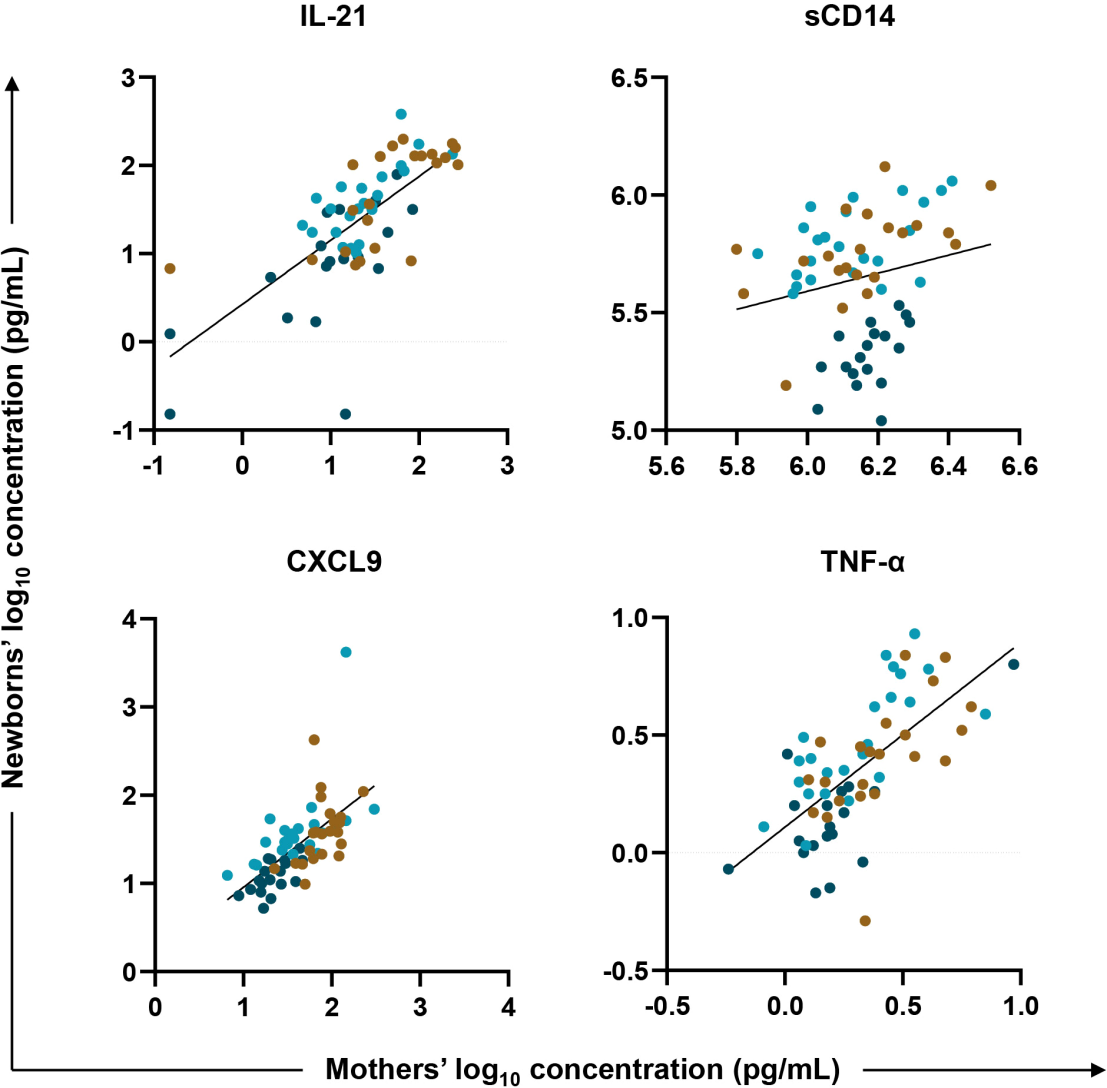
Biomarker correlations between mother/newborn dyads. Linear regression was fit with plasma biomarker concentrations from newborns’ (HUU, HEU-MVS, and HEU-MVNS) values as the outcome (y axis, log_10_ in pg/mL) and their mothers’ (PWOH, PWH-VS and PWH-VNS) values as the variable of interest (x axis, log_10_ in pg/mL) adjusted for mother’s age, newborn’s sex, and mother’s HIV and viral status. Results for four representative biomarkers are shown: IL-21, marker of germinal center development; CXCL9, biomarker of interferon-γ-inducible chemokines; and sCD14 and TNF-α, biomarkers of macrophage activation. Tobit regression was used when data contained values below detection limit, including IL-21. Multiple testing correction was based on p-value < 0.05 and FDR p-value < 0.1. 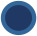 PWOH/HUU; 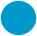 PWH-VS/HEU-MVS; 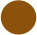 PWH-VNS/HEU-MVNS. PWOH, pregnant without HIV; HUU, HIV-unexposed uninfected; PWH-VS, pregnant with HIV virally suppressed; HEU-MVS, HIV-exposed uninfected born to PWH-VS; PWH-VNS, pregnant with HIV virally non-suppressed; HEU-MVNS, HIV-exposed uninfected born to PWH-VNS.

To test for an association between biomarkers in mother/newborn dyads, a linear regression model fit the newborn (HUU, HEU-MVS, and HEU-MVNS) values as the outcome and maternal (PWOH, PWH-VS and PWH-VNS) values, adjusted for maternal age, newborn sex and maternal HIV status, as the variables of interest. Across all mother/newborn dyads, eleven biomarkers [including GC biomarkers (BAFF, and IL-21), macrophage activation biomarkers (sCD14, and sCD163), T cell differentiation biomarker (IFN-γ, and IL-17A), IFN-γ-inducible chemokine (CXCL9), immune activation chemokines (CCL4, and CCL5), and inflammatory cytokines (TNF-α, and IL-6)] correlated significantly (**Supplementary Table 5**). Four representative biomarkers, IL-21, CXCL9, sCD14, and TNF-α are shown in **Figure 5**. Results suggest that maternal inflammation related to pregnancy is reflected in their newborns’ bioprofiles.

### 3.5 Persistence of immune activation biomarkers over the first 6 months of life in HEU infants

**Figure 6:**
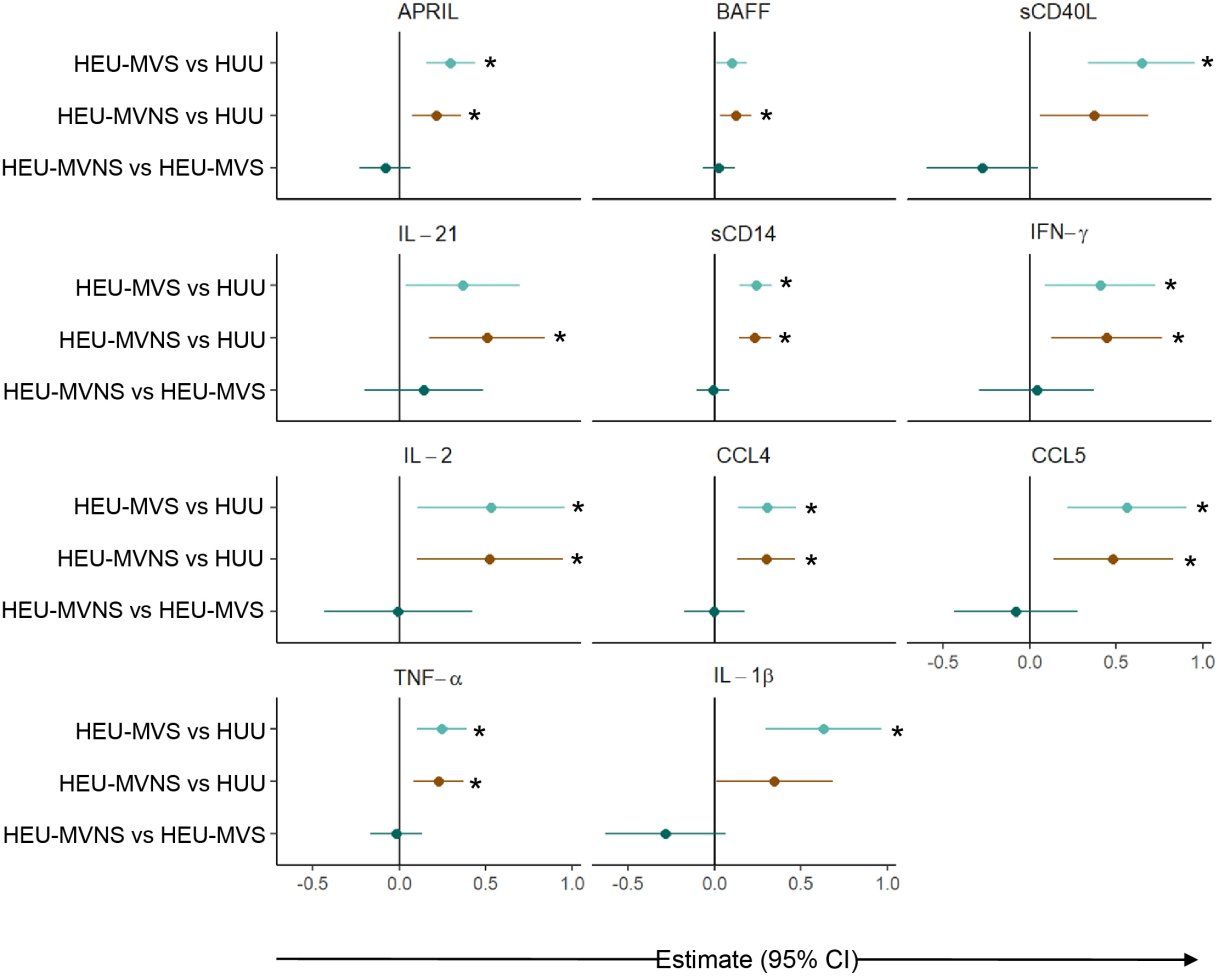
Comparison of plasma biomarker concentrations among infants at 6 months of age. Forest plot for 11 plasma biomarker concentrations which significantly differed among 6-month-old infants without or with exposure to maternal HIV (HUU, HEU-MVS, and HEU-MVNS). The other 10 biomarkers’ concentrations were similar. Pairwise effect estimates and confident intervals are shown from a linear regression model, adjusting for mother’s age and newborn’s sex. Tobit regression was used when data contained values below detection limit, including APRIL, IL-21, IL-2, TNF-α, and IL-1β. For significant biomarkers (un-adjusted p < 0.05 and FDR-adjusted p < 0.1), pairwise comparisons were performed with significance set at p < 0.0167 (p < 0.05/3 Bonferroni correction) and shown with *. 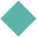 HEU-MVS vs HUU; 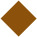 HEU-MVNS vs HUU; 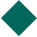 HEU-MVNS vs HEU-MVS. HUU, HIV-unexposed uninfected; HEU-MVS, HIV-exposed uninfected born to virally suppressed mother; HEU-MVNS, HIV-exposed uninfected born to virally non-suppressed mother.

To determine whether the perturbed newborn bioprofiles persisted into infancy, 6-month data from 45 HEU, and 32 HUU infants was analyzed. Similar to the analysis at birth, linear regression analyses, adjusted for maternal age and infant sex, were used to identify 11 biomarkers that were significantly different across all groups (**Supplementary Table 6**). Comparisons of perturbed biomarkers at age 6 months revealed significantly higher concentrations in HEU infants in comparison to HUU infants but no differences between HEU-MVS and HEU-MVNS (**Figure 6**).

**Figure 7:**
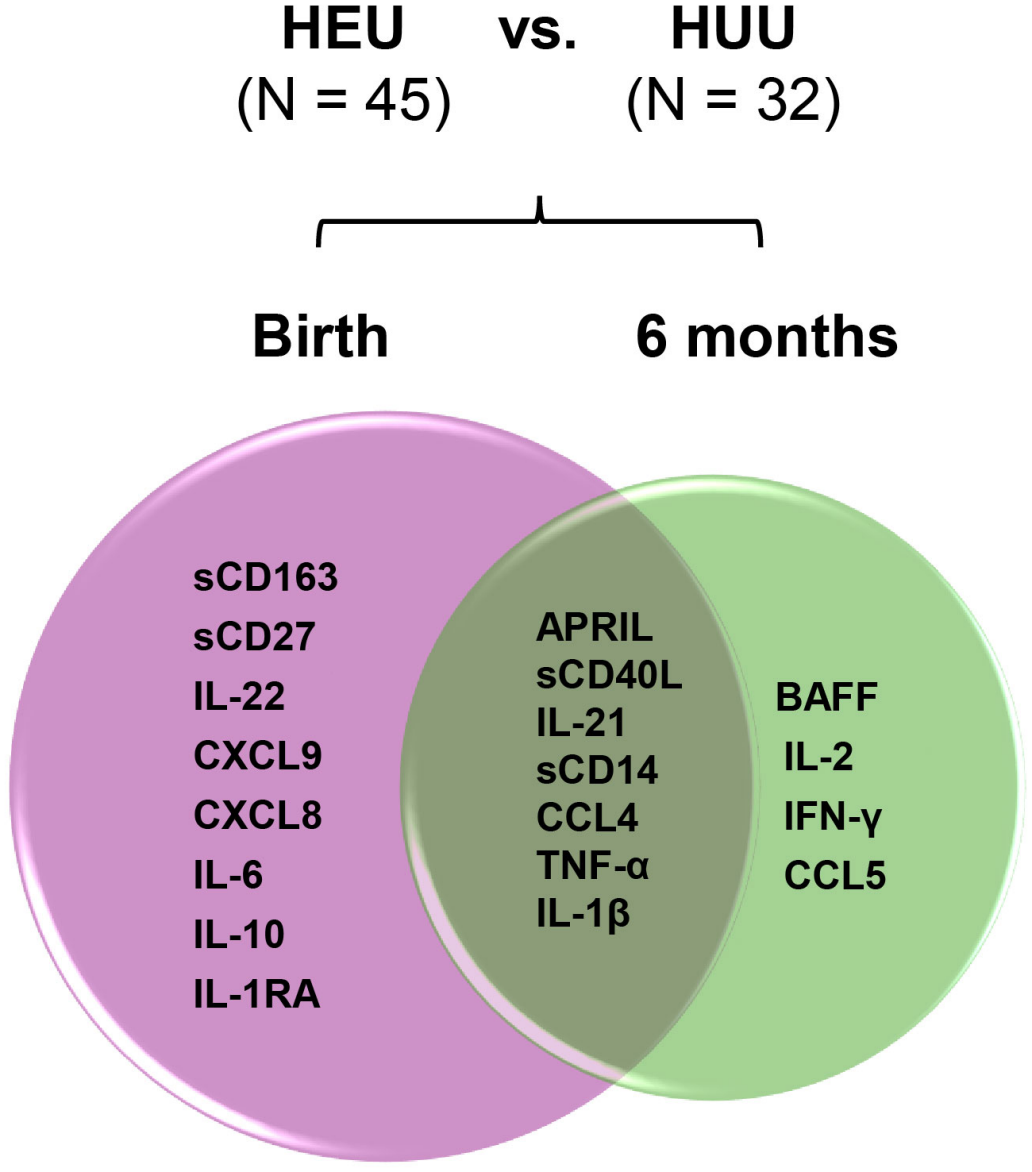
Changes of perturbed biomarkers from birth to 6 months of age in HEU infants in comparison to HUU infants. The plasma biomarkers significantly differed in HEU infants (HEU-MVS plus HEU-MVNS, N = 45) from HUU (N = 32 with longitudinal data) at birth and at 6 months of life were demonstrated by Venn diagram. Biomarkers significantly different by HIV exposure: birth (pink circle) and 6 months (green circle). Biomarkers persistently different at birth and 6 months of life are in the overlapped area. HUU, HIV-unexposed uninfected; HEU, HIV-exposed uninfected; HEU-MVS, HIV-exposed uninfected born to virally suppressed mother; HEU-MVNS, HIV-exposed uninfected born to virally non-suppressed mother.

Bioprofiles in HEU infants in reference to HUU infants changed over time (**Figure 7**). Statistically significant differences at birth between the three groups for sCD163, sCD27, IL-22, CXCL9, CXCL8, IL-6, IL-10, and IL1-RA were no longer evident at 6 months of life. In contrast, birth differences for APRIL, sCD40L, IL-21, sCD14, CCL4, TNF-α, and IL-1β persisted for 6 months, while biomarkers including BAFF, IL-2, IFN-γ, and CCL5 emerged in HEU infants that were significantly different from HUU infants. Changes in plasma concentrations of significant cytokines varied from birth to 6 months. For example, APRIL levels declined from birth to 6 months while sCD14 and TNF-α increased in both HUU and HEU infants (**Supplementary Table 2**). The sensitivity analysis suggested that the results were not changed by feeding methods (data not shown).

### 3.6 Bioprofiles in PWH and HEU newborns are distinct from PWOH and HUU newborns

**Figure 8:**
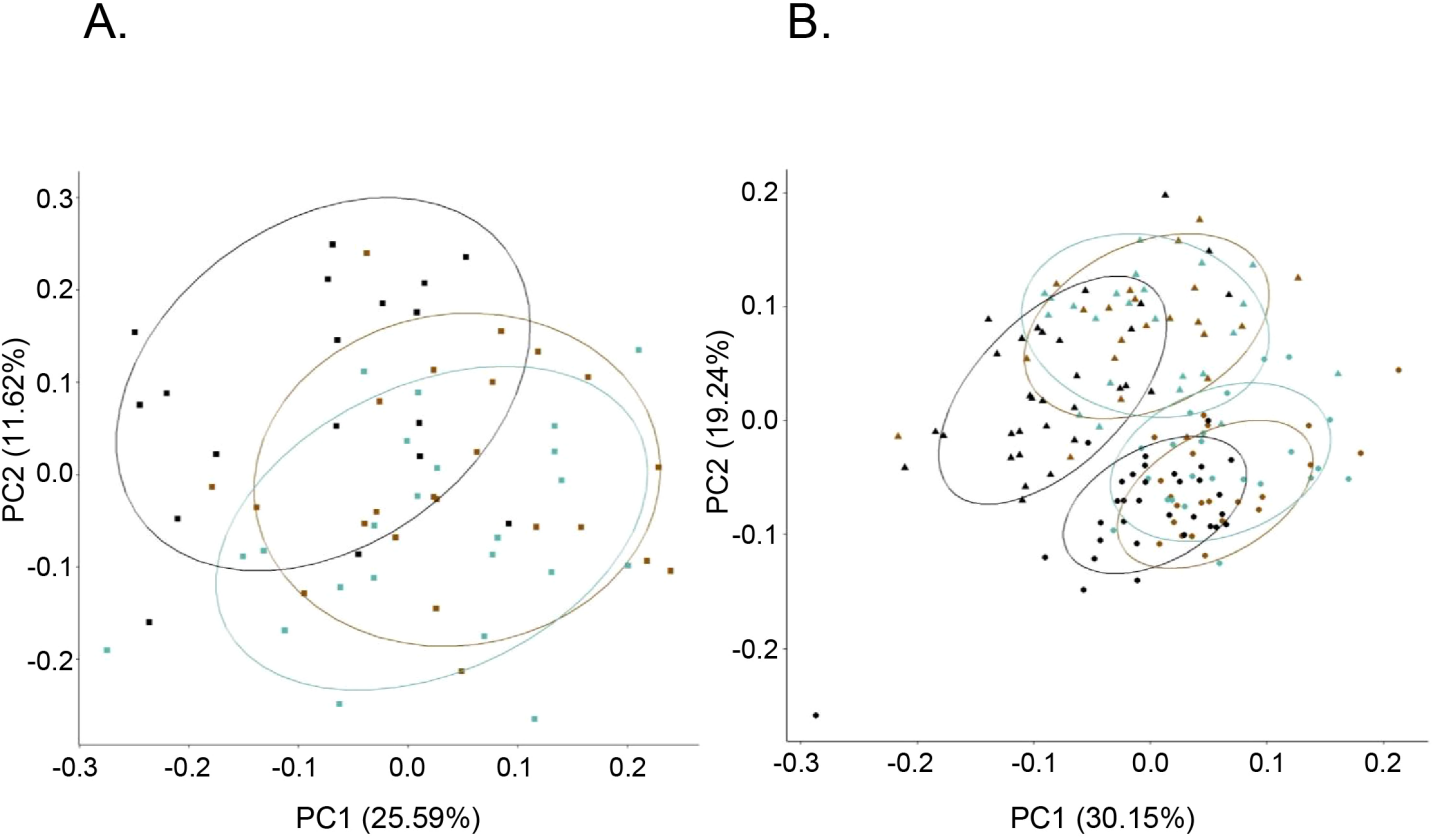
Bioprofile relatedness among pregnant women and among newborns. PCA of pregnant women **(A)**, and newborn and infants **(B)** were generated using the prcomp package in R on the log_10_ transformed biomarker concentrations. **(A)** 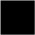 PWOH; 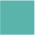 PWH-VS; 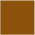 PWH-VNS. **(B)** HUU (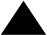 CB; 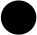 6-months); HEU-MVS (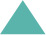 CB; 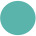 6-months); HEU-MVNS (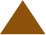 CB; 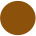 6-months). PCA, principal component analysis; PWOH, pregnant without HIV; PWH-VS, pregnant with HIV virally suppressed; PWH-VNS, pregnant with HIV virally non-suppressed; HUU, HIV-unexposed uninfected; HEU-MVS, HIV-exposed uninfected born to PWH-VS; HEU-MVNS, HIV-exposed uninfected born to PWH-VNS.

Bioprofile relatedness shows considerable overlap between PWH-VS and PWH-VNS, which was distinct from PWOH (**Figure 8A**). In newborns and infants, there was a considerable overlap between HEU infants regardless of their mother’s viral status and both were distinct from HUU. Furthermore, all newborns at birth were distinct from 6 months with HEU infants distinguishable from HUU at both time points (**Figure 8B**).

## 4 Discussion

PACTG 316 was the largest U.S. multi-site clinical trial to prevent perinatal HIV transmission and was uniquely suited for this study as cryopreserved blood samples and clinical outcomes were available from mothers, newborns, and infants through the first 6 months of life (40). For many years, the application of archived clinical and prospectively collected specimens to generate new research questions has been part of the research priorities of HIV/AIDS research agenda (46). Using stored specimens from completed studies enables secondary analyses involving research techniques and discoveries that did not exist when the primary clinical trials were completed (47). Retrospective analysis of clinical and archived laboratory specimens from completed studies is efficient and less costly than developing new clinical trials and can be maximizes resources to address questions of pathogenesis and generate rationale for new interventions built on the original research. A considerable number of studies have demonstrated the validity of using cryopreserved plasma and cell samples that have been stored decades for immune-based assays (48, 49). However, the use of archived specimens has limitations (50). In the case of our study, the ARV used in PACTG 316 differed across time and does not reflect contemporary ARV used for HIV treatment. The PWOH, HUU, and NPWOH specimens were collected as part of different clinical trials and at different geographic sites because PACTG 316 lacked a reference cohort of women and infants without HIV. Additionally, the viral quantitation assays used to define undetectable viral replication (< 400 RNA copies/mL) are less sensitive than current assays that detect virus as low as 20 RNA copies/ml (51). While CD4 T cell counts were similar and clinical co-morbidities such as active infection and AIDS defining illnesses were excluded, PWH participants selected for study may have had occult viral infections such as maternal CMV, that could influence the bioprofiles in the HEU infants (52, 53). The conclusions regarding the effects of HIV infection during pregnancy and in HEU infants are strengthened by the inclusion of cohorts of non-pregnant and pregnant women without HIV and their HUU infants for comparison to PWH, virally suppressed or not suppressed on ARV. All infants, regardless of their maternal HIV status, were term deliveries, so gestational age and complications associated with prematurity were not included in the model. When the potential influences of confounders such as mode of delivery, maternal ARV, and feeding methods were addressed by performing a sensitivity analysis within specific subsets of newborns and infants the results showed no change in the direction of the association for the significant biomarkers. Collectively, these cohorts allowed for clear comparisons to reveal the effects of maternal HIV infection on immune pathways during late pregnancy and in HEU newborns.

Elevated levels of pro-inflammatory cytokines in PWOH are primarily mediated by maternal cells within the uterus and placenta at the fetal/maternal interface (19, 20). Factors that accentuate inflammatory responses, such as LPS, are associated with adverse pregnancy outcomes including pre-term birth and pre-eclampsia (54, 55). Using PWOH as a comparison group, our study dissects HIV effects on both innate and adaptive immune profiles within pregnant women and the impact of HIV exposure for early immune development in their HEU newborns and infants. Rather than focus solely on macrophage activation, the inclusion of biomarkers involved in GC development (APRIL, BAFF, sCD40L, and IL-21) expands the scope of previous studies (26, 27, 56). In addition to pivotal roles in B cell development, Immune mechanisms within GC mediate pregnancy outcomes in both murine and human models (57, 58). Overall, the specific repertoire of plasma biomarkers used in our study can be accurately measured and validated in routine blood testing, supporting their value as outcome measures in clinical trials (59–64).

Proinflammatory biomarkers during late third trimester pregnancy and labor in healthy women trigger compensatory increases in regulatory cytokines that attenuate the inflammatory impact of late pregnancy; for example, lower levels of IL-10 are associated with pre-eclampsia (23, 24, 65–67). As expected in our study, PWOH in late pregnancy, compared to non-pregnant women, displayed higher levels of sCD14, sCD163, and IL-6. A novel finding in PWOH was the association of late third trimester pregnancy with high levels of APRIL, a GC biomarker, and sCD27, a biomarker of T cell activation. In PWOH, pregnancy-related immune activation is countered by elevated immune regulatory cytokines IL-10 and IL-1RA (24). Taken together, PWOH who have term pregnancies display a pro-inflammatory bioprofile characterized by macrophage activation, but also elevated levels APRIL and anti-inflammatory cytokines, all known to play key roles in maintaining normal pregnancies (20, 22–25).

Overall bioprofiles of pregnant women with HIV are distinct from PWOH, although some individual biomarkers are similar. Macrophage activation biomarkers sCD14 and IL-6 are associated with chronic inflammation in people with HIV, either virally suppressed or not suppressed on ARV (45, 68, 69). Yet no significant differences were found in our study between PWOH and PWH. Absence of an association between these macrophage activation biomarkers in pregnancy is likely due to innate immune triggers, particularly through TLR4 during normal labor, that are similar to chronic HIV infection. Intrauterine microbes may activate TLR4 on macrophages during normal pregnancy to induce a macrophage response (25, 43, 54). Furthermore, blockade of TLR4 improves pregnancy outcome in murine models, suggesting that failure to regulate macrophage activation leads to adverse pregnancy outcomes including pre-term birth and pre-eclampsia (70). It remains unclear if a TLR4 inflammatory mechanism plays a role in HIV-associated pregnancy complications (26, 27, 71).

Although longitudinal comparisons of women with HIV before and during pregnancy indicate that CXCL10 and sCD14 levels decline in pregnancy (56), these studies lacked PWOH as a comparison group. Compared to PWOH in our study, PWH showed higher levels of several pro-inflammatory biomarkers, most notably the IFN-γ-inducible chemokine CXCL9, which was further elevated in PWH with detectable viral replication, and the pro-inflammatory chemokine CCL5. These chemokines are known biomarkers of macrophage activation during viral infection, including HIV, Hepatitis C, and SARS-COV2, as well as other inflammatory states associated with macrophage activation (72–77). PWH had lower APRIL levels but no statistical differences in BAFF. Additional studies are needed to determine if levels of APRIL, sCD40L and IL-21, which were higher in PWH compared to PWOH, are associated with adverse pregnancy outcomes in PWH. Within the study cohort the cutoff for undetectable viral suppression was < 400 copies/ml, higher than current viral detection platforms with cutoffs of < 50 copies/ml. However, many studies have demonstrated persistent elevated pro-inflammatory biomarkers even when viral RNA is < 50 copies with ARV (45, 78, 79). Overall, few differences in biomarker profiles between PWH-VS and PWH-VNS indicate that some elevated inflammatory biomarkers in PWH are independent of viral replication.

Previous studies show higher levels of macrophage activation and inflammation in HEU newborns (37). Similarly, HEU newborns in our cohort had elevated sCD14 and IL-6 indicating enhanced macrophage activity at the fetal maternal interface with elevated immune regulatory cytokines to counter immune activation (39). Underdeveloped GC in newborns contribute to the hypo-responsiveness of HUU infants to immunizations in early life (21, 28). Macrophage activation biomarkers in HUU CB, such as sCD14, are associated with higher post-vaccination *pertussis* responses, whereas CB concentrations of APRIL are associated with lower *pertussis* titers after immunization (36). In contrast, CB concentrations of sCD14 and APRIL were positively associated with sustained post-immunization titers to tetanus, demonstrating how different GC bioprofiles are associated with vaccine responsiveness. In addition to biomarkers associated with macrophage activation, HEU newborns in the PACTG 316 cohort, compared to HUU newborns, displayed higher concentrations of GC biomarkers APRIL, sCD40L, and IL-21. Together, PWH and their HEU newborns had elevated proinflammatory bioprofiles including T cell activation and IFN-γ-inducible chemokine such as CXCL9. While HEU infants have higher infectious morbidity and mortality compared to HEU infants, the precise mechanisms are unclear (80). Many studies have shown that passively acquired maternal immunity from PWH is lower compared to PWOH (11, 81, 82). However, results of post immunization immune responses vary among studies of HEU infants and children. Compared to HUU infants, HEU infants have more robust post immunization titers to *pertussis* and conjugated bacterial immunogens but lower tetanus responses (83–85). Disruption of GC dynamics in HEU infants is likely to play a role in determining how HEU infants respond to immunizations based on the nature of the immunogen. GC biomarkers may predict how HEU infants responds to vaccination and explain apparent discrepancies in immunization outcomes to individual vaccines.

Bioprofiles in HEU newborns were distinct from HUU newborns independent of the extent of maternal viral suppression (5, 37), while bioprofiles of HEU and HUU newborns were distinct from their mothers. Regression analysis between all mother/newborn dyads revealed multiple significant associations including GC biomarkers (BAFF and IL-21), macrophage activation (sCD14 and sCD163), cytokines (IFN-γ, IL-17A TNF-α, and IL-6), and chemokines (CXCL9, CCL4, and CCL5). Most evidence indicate that there is little transfer of proinflammatory cytokines across the placenta (86). Fetal/maternal hemorrhage may results in factors within maternal blood that activate immune receptors in the newborn (87, 88). However, based on studies of mother/baby dyads in other viral infections such as SARS CoV2, shared inflammatory triggers effecting both mother and newborns is the most likely explanation in HEU newborns (22, 73, 89). In HEU newborns these shared triggers could include LPS, HIV proteins, other infections, environmental, or genetic factors leading to activation of maternal and fetal immunity through similar pathways (58, 79, 90–93). There is also recent evidence that alterations in maternal immune regulatory function can result in epigenetic changes in CB (94–98). In any case, it is clear that maternal immunity is imprinted on the newborns to shape early immune development (91, 92).

**Figure 9:**
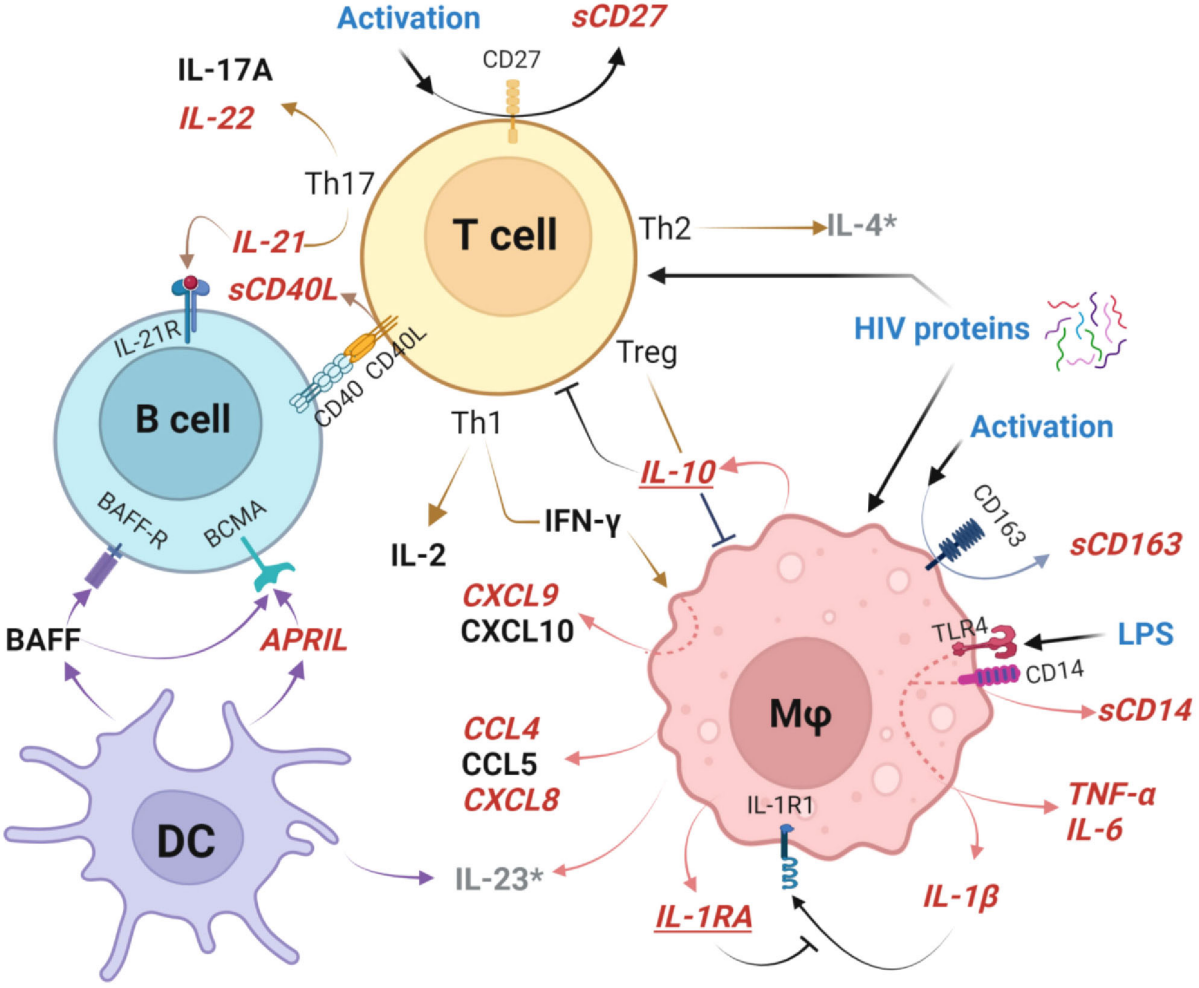
Germinal center cellular networks disrupted in HIV-exposed uninfected newborns. Plasma biomarkers elevated in HEU newborns (HEU-MVS and/or HEU-MVNS), compared to HUU newborns, are shown in bold red italics font. Biomarkers that are similar in HEU and HUU newborns are shown in black font. Elevated cleavage of sCD163 and sCD14 indicate Mφ activation and associated elevated concentrations of TNF-α, IL-6, IL-1β, CCL4, and CXCL8 as well as IFN-γ-inducible chemokine CCXL9. Cleavage of CD27 to sCD27 is a biomarker of T cell activation, with specific increases Th17-associated cytokines IL-22 and IL-21 as well as release of sCD40L at the T cell/B cell interface. DC release higher concentrations of APRIL. Mφ and T cell activation are simultaneously countered by IL-10 from Treg cells, and IL-1RA which blocks IL-1β receptor binding to attenuate multiple cellular inflammatory pathways. These immunoregulatory biomarkers are shown in bold red italics and underlined. Possible triggers of GC activation include LPS activation through TLR4 and/or effects of HIV proteins on newborn cells (54, 79). Biomarkers IL-4 and IL-23 (shown as grey*) were not included in the analysis because > 30% of samples had levels below the limit of detection. This graph was created with BioRender.com. HEU, HIV-exposed uninfected; HEU-MVS, HIV-exposed uninfected born to virally suppressed mother; HEU-MVNS, HIV-exposed uninfected born to virally non-suppressed mother; HUU, HIV-unexposed uninfected; Mφ, macrophage; DC, dendritic cells. Treg, regulatory T cells; GC, germinal center.

Perturbations of GC cellular pathways altered at delivery in HEU newborns are outlined in **Figure 9**. Most notably, TLR4/CD14 signaling results in macrophage activation with subsequent TNF-α, IL-6, IL-1β, CCL4, and CXCL8 secretion (25). The accelerating inflammatory response is associated with shedding of sCD163 and IFN-γ-induced CCXL9 (55, 99). At the same time, HIV-associated T cell activation, manifest by shedding of sCD27, induces IL-22 and IL-21 production which perturbs T cell/B cell interactions through CD40/CD40L altering B cell development (34). Dendritic cells are impacted as evidenced by higher production of APRIL by HEU newborns. These drivers of T cell and macrophage mediated activation are simultaneously countered by production of IL-1RA which blocks IL-1β, as well as production of IL-10 to attenuate multiple cellular inflammatory pathway including those triggered by mediators such as LPS and HIV proteins (69, 79, 100). There is evidence that elevated plasma LPS levels and microbial translocation causes persistent macrophage activation even with optimal control of viral replication by ARV in infected individuals. It is unclear if these factors play a role in the pro-inflammatory bioprofiles of HEU infants (68, 69, 75, 79, 90, 100).

Normalization of many inflammatory biomarker levels by immune regulatory mechanisms may suggest a transient maternal effect on HEU infant immune development. However, several biomarkers, including APRIL, sCD40L and IL-21, remained perturbed in HEU infants at 6 months suggesting a sustained effect on GC pathways that could affect B cell development and immunoglobulin class switch. By 6 months of age emergence of perturbations in the plasma concentration of BAFF, T cell chemoattractant CCL5, and Th1 cytokines IL-2 and IFN-γ support the concept that HEU infants have persistent selective alterations in immunity throughout infancy (38). Application of these biomarkers, when linked to long-term clinical outcomes in HEU infants and children, may provide further insight into the long-term complications associated with children born to women living with HIV. The fetal/maternal interface lies at the nexus of innate and adaptive immunity, to shape GC development. There is increasing evidence that the intrauterine environment not only affects pregnancy outcomes but also primes infant immunity to increase the risk for allergic disorders and the effectiveness of vaccine responses (81, 82, 84, 93, 101, 102). Our results indicate that immune regulatory responses down-regulate inflammation and are likely to preserve immune function in HEU infants. However, there may be subsets of HEU infants who are at risk for long-term infections and immune dysregulation as a result of maternal HIV infection (13). Extended longitudinal studies of bioprofiles of immune networks are needed to assess the long-term health consequences from gestational viral exposure in HEU infants.

## Data Availability

The original contributions presented in the study are included in the article/**Supplementary Material**. Further inquiries can be directed to the corresponding author.
