# Supplementary material for: Maternal immunity shapes biomarkers of germinal center development in HIV‐exposed uninfected infants
Source: Front Immunol. 2024 Sep 12;15:1443886. doi: 10.3389/fimmu.2024.1443886 (PMC11424517; doi:10.3389/fimmu.2024.1443886)
Supplement: Supplementary file 1 [file DataSheet1.docx]

| **Function** | **Biomarker** | **Plasma biomarker concentration** [median (25%-75% quartile range)] (pg/mL) | | | |
| --- | --- | --- | --- | --- | --- |
|  |  | **NPWOH (N = 21)** | **PWOH (N = 18)** | **PWH-VS (N = 23)** | **PWH-VNS (N = 23)** |
| **B cell and GC  development** | **APRIL** | **31,724** (25,426 - 36,633) | **70,788** (58,063 - 91,697) | **27,499** (15,769 - 40,878) | **40,078** (24,802 - 52,344) |
|  | **BAFF** | **666** (488 - 809) | **663** (567 - 878) | **634** (401 - 772) | **604** (513 - 751) |
|  | **sCD40L** | **218** (137 - 402) | **81** (56 - 156) | **392** (187 - 673) | **312** (129 - 501) |
|  | **IL-21** | **12.8** (8.8 - 25.4) | **11.3**  (5.9 - 32.9) | **20.8** (13.0 - 38.3) | **36.2** (17.8 - 140.7) |
| **Macrophage**  **activation** | **sCD14** | **871,243** (556,420 -  1,290,880) | **1,494,209** (1,328,937 -  1,696,146) | **1,288,020** (1,013,650 -  1,875,541) | **1,391,720** (1,158,090 -  1,708,621) |
|  | **sCD163** | **275,818** (203,144 - 453,676) | **463,314** (311,619 - 699,169) | **571,687** (427,704 - 755,053) | **757,147** (516,994 - 996,131) |
| **T cell activation**  **and differentiation** | **sCD27** | **3,592** (3,033 - 5,285) | **4,953** (3,550 - 7,180) | **7,903** (4,416 - 11,666) | **8,735** (5,892 - 15,053) |
|  | **IFN-γ** | **3.26** (1.90 - 6.27) | **3.30** (1.31 - 5.14) | **3.19** (1.88 - 5.47) | **4.15** (1.81 - 6.81) |
|  | **IL-17A** | **1.16** (0.61 - 1.54) | **0.89** (0.39 - 1.58) | **1.19** (0.27 - 2.79) | **1.52** (1.00 - 2.2) |
|  | **IL-22** | **0.71** (0.58 - 1.06) | **0.48** (0.32 - 1.18) | **1.17** （0.66 - 1.99） | **1.52** （0.82 - 2.39） |
|  | **IL-2** | **0.51** (0.16 - 1.76) | **0.56** (0.05 - 1.44) | **0.67** （0.41 - 1.36） | **0.63** （0.33 - 1.28） |
| **IFN-γ-inducible  chemokines** | **CXCL9** | **21.90** (16.19 - 39.50) | **20.20** (16.16 - 29.61) | **36.66** （27.67 - 69.17） | **76.39** （56.03 - 117.97） |
|  | **CXCL10** | **182** (168 - 334) | **239** (173 - 432) | **291** （157 - 490） | **457** （375 - 638） |
| **Immune activation**  **chemokines** | **CCL4** | **44.83** (35.56 - 62.86) | **56.00** (32.35 - 80.26) | **60.45** （51.67 - 104.22） | **60.00** （42.07 - 84.86） |
|  | **CCL5** | **53,544** (22,470 - 72,132) | **15,039** (9,243 - 29,545) | **50,524** （32,432 - 136,016） | **55,023** (22,016 - 110,444) |
|  | **CXCL8** | **2.52** (1.65 - 4.25) | **2.06** (1.37 - 3.53) | **3.27** (2.06 - 8.52) | **3.65** (1.82 - 6.46) |
| **Inflammatory**  **cytokines** | **TNF-α** | **1.03** (0.89 - 1.69) | **1.53** (1.18 - 1.79) | **2.16** (1.25 - 2.89) | **2.29** (1.51 - 3.58) |
|  | **IL-1β** | **0.25** (0.04 - 0.49) | **0.09** (0.01 - 0.27) | **0.43** (0.25 - 1.06) | **0.18** (0.06 - 0.51) |
|  | **IL-6** | **1.28** (0.83 - 1.97) | **2.73** (1.57 - 6.72) | **2.15** (1.20 - 7.34) | **3.12** (1.98 - 4.66) |
| **Anti-inflammatory/ immune regulatory**  **cytokines** | **IL-10** | **0.15** (0.08 - 0.25) | **0.29** (0.17 - 0.40) | **0.35** (0.18 - 1.54) | **0.38** (0.26 - 0.50) |
|  | **IL-1RA** | **162** (114 - 285) | **329** (237 - 577) | **532** (270 - 664) | **418** (266 - 574) |

**Table S1. Plasma biomarker concentrations in non-pregnant and pregnant women without HIV, and pregnant women with HIV virally suppressed or non-suppressed.**

**Abbreviations.** NPWOH, non-pregnant without HIV; PWOH, pregnant without HIV; PWH-VS, pregnant with HIV virally suppressed; PWH-VNS: pregnant with HIV virally non-suppressed; GC, germinal center.

**Table S2. Plasma biomarker concentrations in HIV-exposed uninfected and HIV-unexposed uninfected infants at birth and at 6 months of life.**

| **Function** | **Biomarker** | **Plasma biomarker concentration** [median (25%-75% quartile range)] (pg/mL) | | | | | |
| --- | --- | --- | --- | --- | --- | --- | --- |
|  |  | **At birth (CB)** | | | **At 6 months of life** | | |
|  |  | **HUU**  **(N = 50)** | **HEU-MVS**  **(N = 23)** | **HEU-MVNS**  **(N = 22)** | **HUU**  **(N = 32)** | **HEU-MVS**  **(N = 23)** | **HEU-MVNS**  **(N = 22)** |
| **B cell and GC  development** | **APRIL** | **41,174** (32,402 -  59,221) | **75,003** (61,106 -  126,971) | **90,498** (68,645 -  130,131) | **18,845** (14,458 -  25,557) | **30,913** (21,254 -  51,116) | **26,982** (18,802 –  42,360) |
|  | **BAFF** | **1,306** (950 -  2,595) | **3,052** (1,624 -  4,774) | **3,188** (1,491 -  3,938) | **696** (613 -  868) | **913** (787 -  1,164) | **996** (740 -  1,262) |
|  | **sCD40L** | **320** (176 - 479) | **731** (264 - 1,229) | **576** (225 - 1,418) | **263** (119 - 369) | **780** (352 - 1,437) | **508** (152 - 1,334) |
|  | **IL-21** | **17** (7 - 35) | **34** (18 - 74) | **101** (10 - 131) | **19** (7 - 37) | **27** (17 - 80) | **42** (21 - 170) |
| **Macrophage  activation** | **sCD14** | **253,093** (179,812 - 311,029) | **598,552** (457,122 -  881,055) | **591,045** (450,170 -  766,562) | **957,688** (750,842 -  1,312,121) | **1,457,144** (1,211,222 -  1,971,140) | **1,539,803** (1,229,151 -  1,811,648) |
|  | **sCD163** | **325,569** (212,634 -  481,415) | **442,387** (327,320 -  663,492) | **374,893** (274,011 -  594,595) | **480,980** (379,500 –  792,085) | **482,121** (377,030 –  725,022) | **590,498** (428,446 –  1,060,278) |
| **T cell activation and  differentiation** | **sCD27** | **4,768** (3,489 -  7,201) | **8,470** (6,663 -  10,682) | **6,735** (4,237 -  9,032) | **9,831** (8,062 -  11,518) | **11,393** (10,097 -  14,230) | **10,831** (9,662 -  16,699) |
|  | **IFN-γ** | **0.76** (0.10 - 1.60) | **1.80** (0.41 - 3.14) | **1.37** (0.10 - 2.92) | **5.58** (2.84 - 10.87) | **8.68** (4.58 - 14.19) | **8.45** (4.29 - 19.32) |
|  | **IL-17A** | **0.39** (0.02 - 0.80) | **0.70** (0.02 - 1.82) | **0.87** (0.02 - 1.73) | **2.55** (1.83 - 4.69) | **3.30** (1.82 - 6.75) | **3.18** (2.05 - 7.28) |
|  | **IL-22** | **0.31** (0.16 - 0.51) | **0.63** (0.27 - 1.01) | **0.62** (0.34 - 1.06) | **2.00** (1.45 - 3.48) | **3.41** (2.16 - 8.21) | **4.04** (2.98 - 5.02) |
|  | **IL-2** | **0.17** (0.05 - 0.48) | **0.42** (0.05 - 1.23) | **0.52** (0.05 - 1.26) | **0.35** (0.05 - 1.25) | **0.80** (0.39 - 1.99) | **0.92** (0.54 - 1.79) |
| **IFN-γ-inducible  chemokines** | **CXCL9** | **15.7** (10.5 - 26.2) | **32.7** (24.0 - 48.4) | **37.5** (20.0 - 57.7) | **50.9** (36.2 - 69.5) | **52.6** (39.3 - 105.4) | **71.3** (50.1 - 110.0) |
|  | **CXCL10** | **148** (81 - 227) | **124** (70 - 315) | **135** (94 - 199) | **250** (172 - 370) | **342** (206 - 779) | **322** (204 - 721) |
| **Immune activation chemokines** | **CCL4** | **76** (57 - 117) | **217** (151 - 337) | **252** (161 - 324) | **95** (62 - 139) | **193** (130 - 216) | **227** (118 - 331) |
|  | **CCL5** | **42,799** (16,679 -  85,513) | **84,742** (26,230 -  133,725) | **45,955** (18,339 -  240,402) | **71,457** (40,531 -  102,717) | **147,176** (87,434 -  295,305) | **150,071** (49,379 -  269,023) |
|  | **CXCL8** | **10.40** (4.32 - 5.76) | **6.44** (2.66 - 11.30) | **15.62** (8.14 - 67.87) | **8.70** (4.58 - 16.31) | **7.55** (5.44 - 16.96) | **8.51** (4.14 - 14.57) |
| **Inflammatory  cytokines** | **TNF-α** | **1.57** (1.13 - 2.05) | **2.61** (2.01 - 4.57) | **2.58** (1.77 - 3.35) | **2.80** (2.10 - 4.37) | **4.53** (3.44 - 8.38) | **3.98** (3.44 - 7.48) |
|  | **IL-1β** | **0.68** (0.20 - 1.87) | **2.16** (0.66 - 3.57) | **1.17** (0.57 - 2.92) | **0.18** (0.09 - 0.31) | **0.60** (0.17 - 1.77) | **0.32** (0.16 - 1.05) |
|  | **IL-6** | **1.73** (1.22 - 3.41) | **6.86** (1.84 - 21.31) | **3.33** (1.57 – 18.38) | **1.28** (0.92 - 2.22) | **2.20** (1.16 - 3.40) | **1.85** (1.02 – 2.59) |
| **Anti-inflammatory/ immune regulatory cytokines** | **IL-10** | **0.24** (0.14 - 0.48) | **1.94** (0.87 - 4.58) | **1.19** (0.55 - 3.19) | **0.80** (0.41 - 1.19) | **0.88** (0.57 - 1.53) | **0.93** (0.50 - 1.45) |
|  | **IL-1RA** | **474** (232 - 1,005) | **2,979** (1,076 - 5,815) | **1,407** (582 - 2,752) | **193** (154 - 409) | **494** (186 - 803) | **273** (166 - 424) |

**Abbreviations.** CB, cord blood; HUU, HIV-unexposed uninfected; HEU-MVS, HIV-exposed uninfected born to virally suppressed mother; HEU-MVNS, HIV-exposed uninfected born to virally non-suppressed mother; GC, germinal center.

**Table S3. Comparison of plasma biomarker concentrations among pregnant**

**women without HIV and pregnant women with HIV (virally suppressed**

**or virally non-suppressed).**

| **Function** | **Biomarker** | **P value** | **FDR P value** |
| --- | --- | --- | --- |
| **B cell and germinal center (GC) development** | **APRIL*** | **0.000256** | **0.00134** |
|  | BAFF | 0.438 | 0.613 |
|  | **sCD40L** | **7.45e-06** | **7.82e-05** |
|  | **IL-21*** | **0.00489** | **0.0128** |
| **Macrophage  activation** | sCD14 | 0.659 | 0.728 |
|  | **sCD163** | **0.00472** | **0.0128** |
| **T cell activation and  differentiation** | **sCD27** | **0.000613** | **0.00257** |
|  | IFN-γ* | 0.717 | 0.753 |
|  | IL-17A* | 0.151 | 0.289 |
|  | IL-22 | 0.236 | 0.381 |
|  | IL-2* | 0.648 | 0.728 |
| **IFN-γ-inducible  chemokines** | **CXCL9** | **7.09e-07** | **1.49e-05** |
|  | **CXCL10*** | **0.0287** | **0.061** |
| **Immune activation chemokines** | CCL4 | 0.186 | 0.325 |
|  | **CCL5** | **0.000134** | **0.000941** |
|  | CXCL8 | 0.479 | 0.628 |
| **Inflammatory  cytokines** | **TNF-α** | **0.0291** | **0.061** |
|  | **IL-1β*** | **0.00403** | **0.0128** |
|  | IL-6 | 0.795 | 0.795 |
| **Anti-inflammatory/ immune regulatory cytokines** | IL-10 | 0.349 | 0.524 |
|  | IL-1RA | 0.573 | 0.708 |

Linear regression with log_10_ of biomarker value as the outcome while adjusting

for the covariate of age.

Bold indicates P-value < 0.05 and FDR p-value < 0.1.

*: Results from Tobit regression.

**Table S4. Comparison of plasma biomarker concentrations among newborns**

**including HIV-unexposed uninfected and HIV-exposed uninfected born to**

**virally suppressed or virally non-suppressed mother.**

| **Function** | **Biomarker** | **P value** | **FDR P value** |
| --- | --- | --- | --- |
| **B cell and germinal  center (GC)  development** | **APRIL** | **1.79e-09** | **1.26e-08** |
|  | BAFF* | 0.141 | 0.165 |
|  | **sCD40L** | **0.049** | **0.0686** |
|  | **IL-21*** | **0.000101** | **0.000302** |
| **Macrophage  activation** | **sCD14** | **6.06e-18** | **1.27e-16** |
|  | **sCD163** | **0.0261** | **0.0421** |
| **T cell activation and  differentiation** | **sCD27** | **0.000198** | **0.00052** |
|  | IFN-γ* | 0.192 | 0.213 |
|  | IL-17A* | 0.408 | 0.428 |
|  | **IL-22*** | **0.00624** | **0.0119** |
|  | IL-2* | 0.0707 | 0.0928 |
| **IFN-γ-inducible  chemokines** | **CXCL9** | **0.000076** | **0.000264** |
|  | CXCL10 | 0.902 | 0.902 |
| **immune activation chemokines** | **CCL4** | **3.5e-09** | **1.84e-08** |
|  | CCL5 | 0.134 | 0.165 |
|  | **CXCL8** | **0.0107** | **0.0188** |
| **Inflammatory  cytokines** | **TNF-α** | **0.00156** | **0.00363** |
|  | **IL-1β*** | **0.0478** | **0.0686** |
|  | **IL-6** | **0.00384** | **0.00807** |
| **Anti-inflammatory/ immune regulatory cytokines** | **IL-10*** | **2.29e-10** | **2.4e-09** |
|  | **IL-1RA** | **2.8e-07** | **1.18e-06** |

Linear regression with log_10_ of biomarker value as the outcome, while adjusting

for the covariate of mother’s age and infant’s gender.

Bold indicates P-value < 0.05 and FDR p-value < 0.1.

*: Results from Tobit regression.

**Table S5. Biomarker correlations between mother/newborn dyads.**

| **Function** | **Biomarker** | **Effect Estimate (95% CI)** | **P value** | **FDR P value** |
| --- | --- | --- | --- | --- |
| **B cell and germinal  center (GC)  development** | APRIL | 0.048 (-0.098; 0.194) | 0.511 | 0.564 |
|  | **BAFF*** | **1.130 (0.458; 1.790)** | **0.000949** | **0.00285** |
|  | sCD40L | 0.221 (-0.160; 0.603) | 0.25 | 0.328 |
|  | **IL-21*** | **0.656 (0.481; 0.831)** | **2.13e-13** | **4.47e-12** |
| **Macrophage  activation** | **sCD14** | **0.619 (0.351; 0.886)** | **0.000022** | **0.000097** |
|  | **sCD163** | **0.478 (0.228; 0.728)** | **0.000328** | **0.00115** |
| **T cell activation and  differentiation** | sCD27 | 0.141 (-0.086; 0.369) | 0.219 | 0.309 |
|  | **IFN-γ*** | **0.492 (0.081; 0.903)** | **0.019** | **0.0399** |
|  | **IL-17A*** | **0.991 (0.543; 1.440)** | **0.000015** | **0.000097** |
|  | IL-22* | 0.140 (-0.084; 0.365) | 0.22 | 0.309 |
|  | IL-2* | 0.142 (-0.180; 0.465) | 0.386 | 0.451 |
| **IFN-γ-inducible  chemokines** | **CXCL9** | **0.682 (0.386; 0.978)** | **0.000023** | **0.000097** |
|  | CXCL10 | 0.015 (-0.266; 0.295) | 0.916 | 0.916 |
| **Immune activation chemokines** | **CCL4** | **0.350 (0.094; 0.607)** | **0.0083** | **0.0218** |
|  | **CCL5** | **0.325 (0.037; 0.613)** | **0.0276** | **0.0528** |
|  | CXCL8 | 0.090 (-0.234; 0.413) | 0.581 | 0.61 |
| **Inflammatory  cytokines** | **TNF-α** | **0.690 (0.480; 0.899)** | **1.49e-08** | **1.57e-07** |
|  | IL-1β* | 0.174 (-0.030; 0.379) | 0.0944 | 0.165 |
|  | **IL-6** | **0.410 (0.075; 0.745)** | **0.0173** | **0.0399** |
| **Anti-inflammatory/ immune regulatory cytokines** | IL-10* | 0.170 (-0.140; 0.480) | 0.281 | 0.347 |
|  | IL-1RA | 0.284 (-0.129; 0.697) | 0.174 | 0.281 |

Linear regression was fit between all mothers (pregnant without HIV, and pregnant with HIV virally suppressed and virally non-suppressed) values and all their corresponding newborns (HIV-unexposed uninfected, and HIV-exposed uninfected born to virally suppressed and virally non-suppressed mother) with log_10_ of biomarker value as the outcome, while adjusting for the covariates of mother’s age, infant’s gender, and the mother’s health status.

Bold indicates P-value < 0.05 and FDR p-value < 0.1.

*: Results from Tobit regression.

**Table S6. Comparison of biomarker concentrations among infants at 6 months of age,**

**including HIV-unexposed uninfected and HIV-exposed uninfected born to virally suppressed**

**or virally non-suppressed mothers.**

| **Function** | **Biomarker** | **P value** | **FDR P value** |
| --- | --- | --- | --- |
| **B cell and germinal  center (GC)  development** | **APRIL*** | **0.000258** | **0.00206** |
|  | **BAFF** | **0.0186** | **0.039** |
|  | **sCD40L** | **0.000388** | **0.00206** |
|  | **IL-21*** | **0.0108** | **0.0285** |
| **Macrophage  activation** | **sCD14** | **6.35e-07** | **1.33e-05** |
|  | sCD163 | 0.377 | 0.396 |
| **T cell activation and  differentiation** | sCD27 | 0.112 | 0.18 |
|  | **IFN-γ*** | **0.0124** | **0.0289** |
|  | IL-17A* | 0.374 | 0.396 |
|  | IL-22 | 0.122 | 0.184 |
|  | **IL-2*** | **0.0206** | **0.0394** |
| **IFN-γ-inducible  chemokines** | CXCL9 | 0.259 | 0.34 |
|  | CXCL10* | 0.303 | 0.375 |
| **Immune activation chemokines** | **CCL4** | **0.000392** | **0.00206** |
|  | **CCL5** | **0.00301** | **0.00903** |
|  | CXCL8* | 0.915 | 0.915 |
| **Inflammatory  cytokines** | **TNF-α*** | **0.00157** | **0.00646** |
|  | **IL-1β*** | **0.00185** | **0.00646** |
|  | IL-6* | 0.325 | 0.379 |
| **Anti-inflammatory/ immune regulatory cytokines** | IL-10* | 0.199 | 0.279 |
|  | IL-1RA | 0.0576 | 0.101 |

Linear regression with log_10_ of biomarker value as the outcome while adjusting for the

covariate of mother’s age and infant’s gender.

Bold indicates P-value < 0.05 and FDR p-value < 0.1.

*: Results from Tobit regression.


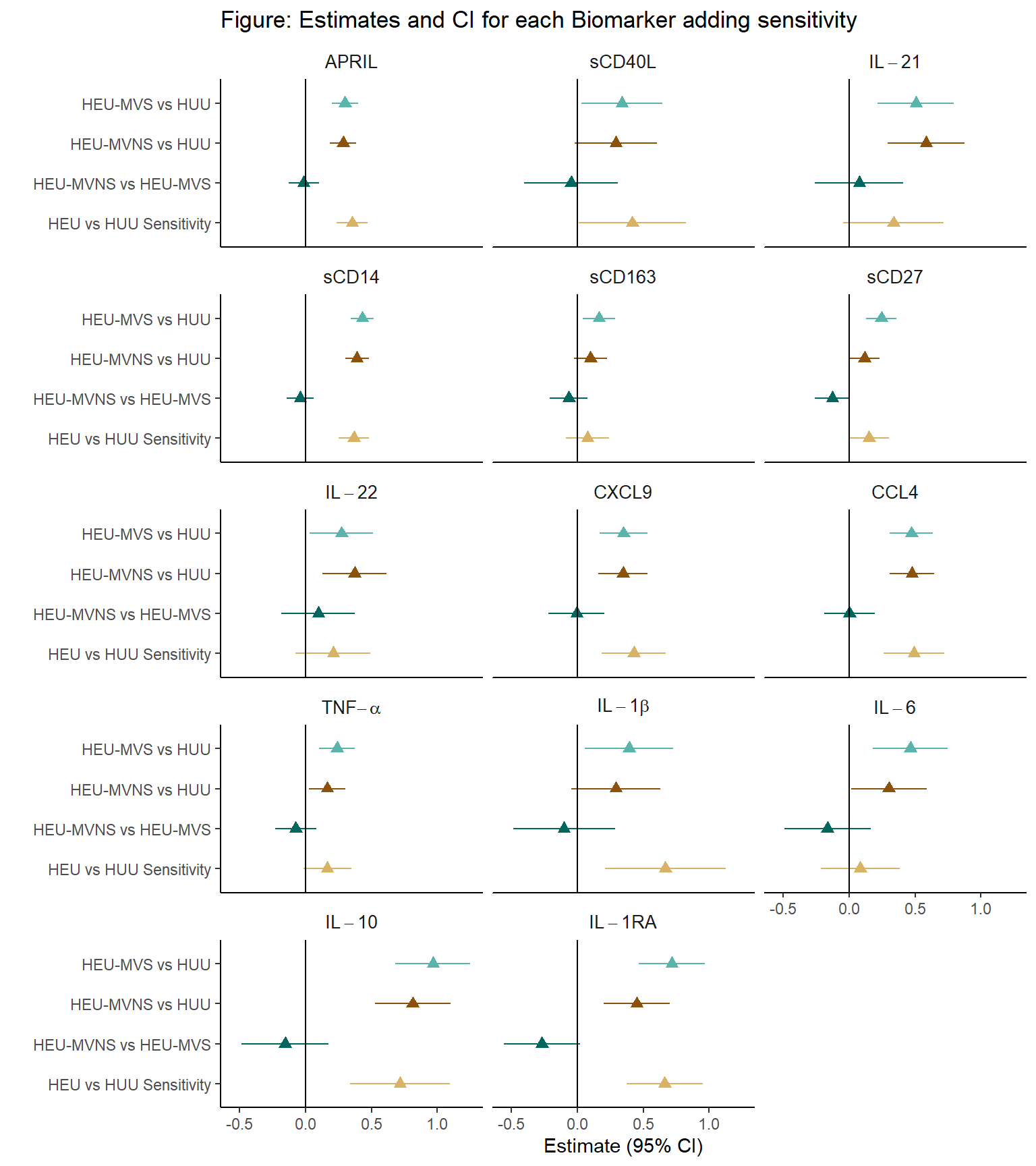


**Figure S1. The effect of delivery mode on biomarker profile in HEU newborn.** The effect of mode of delivery (vaginal or cesarean) on biomarker profile was performed by comparing vaginally born HEU newborns who received placebo at birth (N = 12) to HUU newborns who were all vaginally born (N = 50) [▲ HEU vaginally born taking placebo vs. HUU vaginally delivered] on 14 biomarkers which differed significantly between HUU and HEU newborns (**see Figure 4**) [▲HEU-MVS vs HUU; ▲HEU-MVNS vs HUU; ▲HEU-MVNS vs HEU-MVS]. The delivery mode did not change the direction of the biomarker difference between HEU and HUU without/with delivery mode taken into consideration.
